# An Improved Gold(I) Catalytic System for the Preparation of Coumarins *via* Intramolecular Cyclization

**DOI:** 10.1002/asia.202400725

**Published:** 2024-11-09

**Authors:** Francesco Ravera, Federico Floreani, Cristina Tubaro, Marco Roverso, Riccardo Pedrazzani, Marco Bandini, Andrea Biffis

**Affiliations:** ^1^ Department of Chemical Sciences–DiSC University of Padova Via Marzolo 1 35131 Padova Italy; ^2^ Consorzio Interuniversitario per le Reattività Chimiche e la Catalisi (CIRCC) University of Padova Via Marzolo 1 35131 Padova Italy; ^3^ Department of Chemistry “G. Ciamician” Alma Mater Studiorum − University of Bologna Via Selmi 2 I-40126 Bologna Italy

**Keywords:** Gold, Coumarin, Hydroarylation, Cyclization, Ionic liquids

## Abstract

A catalytic system comprising a gold(I) complex with an N‐heterocyclic carbene (NHC) ligand in an ionic liquid as solvent exhibits higher catalytic efficiency compared to state of the art systems in the title reaction, which enables using down to 0.01 mol % gold. A commercial gold(I) catalyst such as IPrAuNTf_2_ can be employed for this purpose. In the case of less reactive substrates bearing electron‐withdrawing substituents at the phenol moiety, a tailor made NHC‐gold(I) precatalyst exhibits improved reactivity and can be advantageously employed compared to the commercial one.

## Graphical abstract

A new catalytic system based on a gold(I) complex in a small amount of a suitable ionic liquid was reported, showing improved performances in gold‐catalyzed intramolecular hydroarylations of aryl alkynoates to yield coumarins. Very low catalyst loadings could be employed in case of activated substrates with a good tolerance toward substituents, allowing to access a variety of susbtituted coumarins.

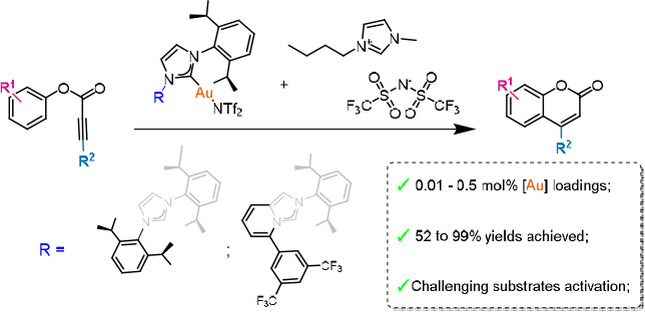

## Introduction

**Scheme 1:**
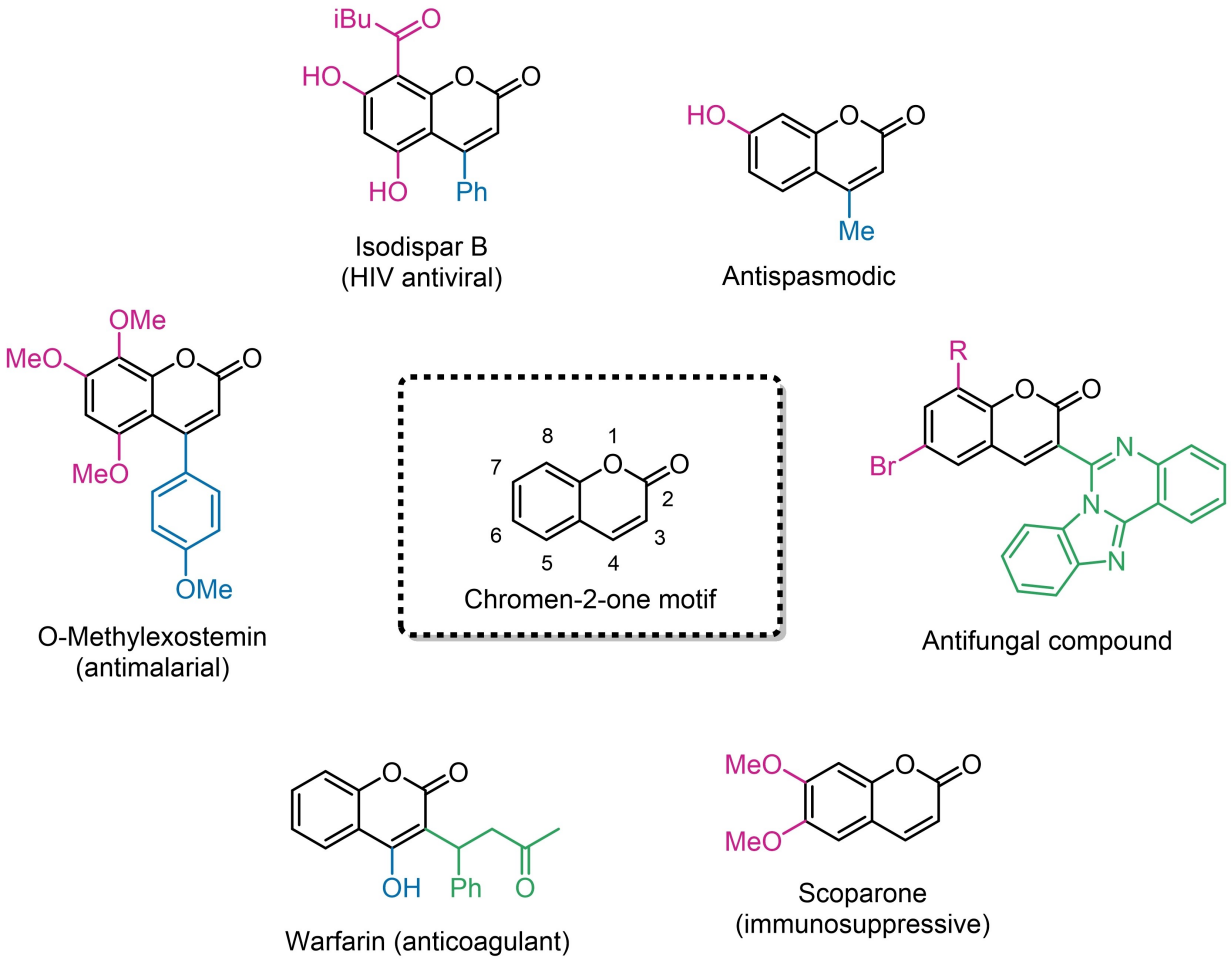
Selection of bioactive compounds based on the coumarin core.

Coumarins are heterocyclic molecules characterized by a structure (the chromen‐2‐one motif) which is widespread over many natural products (Scheme 1).[^1^, ^2^, ^3^, ^4^, ^5^, ^6^] Most of them exhibit biological activity (anticancer,^[7]^ anti‐inflammatory,[^8^, ^9^] antifungal,^[10]^ etc) and/or good luminescence properties for photoelectronic applications,[^11^, ^12^, ^13^] depending on the substituents decorating the bicyclic backbone of the molecule.[^7^, ^14^, ^15^]

A large series of synthetic approaches based on stoichiometric organic reactions were consequently developed to build and edit the structure of chromen‐2‐ones in order to subsequently explore and exploit the different properties of this family of heterocycles. However, traditional strategies such as the Pechmann condensation or the Perkin synthesis require harsh conditions and often lead to low yields and poor atom economy of the process. More modern synthetic approaches employ π‐acidic transition metal catalysts (Pd, Pt, Au) to activate the double or triple bond in phenol‐derived acrylates or propiolates, consequently triggering the cyclization at the aromatic ring.[^15^, ^16^, ^17^, ^18^, ^19^, ^20^]

In particular, gold(I)‐based complexes have emerged as promising precatalysts for this task,[^21^, ^22^] enabling a simple two steps synthesis of coumarins, which consists in a pre‐esterification step of a phenol with a propiolic acid, mediated by a di‐substituted carbodiimide, followed by an intramolecular hydroarylation process started by coordination of the alkyne at gold.[^23^, ^24^, ^25^, ^26^, ^27^, ^28^, ^29^, ^30^, ^31^, ^32^, ^33^, ^34^, ^35^, ^36^]

In this respect, a former report by Banwell et al. showcases the main trends and limitations of gold(I) precatalysts in the cyclization of aryl propiolate derivatives.^[36]^ The appropriate activation of the aromatic ring toward electrophilic substitution by derivatization with electron donating functional groups is apparently crucial to determine good catalytic performances. Moreover, relatively high catalyst loadings are required (3–5 mol %), which represents a major limitation in gold catalysis.[^37^, ^38^] Indeed, if other gold‐catalyzed hydrofunctionalization processes (e. g. alkyne hydration, hydroamination, hydroalkoxlation) are known to work efficiently with amounts of gold that can reach very low values (down to<0.1 mol %),^[39]^ only few examples are reported with respect to hydroarylation reactions.^[40]^

Under this framework, the demand for a new efficient catalytic protocol for this reaction, capable of merging lower working gold loadings and broader substrate scope, is urgent. Our longstanding experience in the study of late transition metal catalysts for direct alkyne hydroarylation^[41]^ reactions led us recently to develop a system based on gold(I) complexes in ionic liquids (ILs) as catalysts featuring high activity in the hydroarylation of alkynoic acids and esters with electron rich arenes and heteroarenes under mild conditions.[^42^, ^43^] We then set out to apply this reaction to the development of a synthetic protocol for the preparation of functionalized coumarins via intramolecular hydroarylation of phenol derived propiolic esters.

## Results and Discussion

Our investigation started with the preparation of a benchmark substrate **1 a** to screen the best conditions to perform the intramolecular hydroarylation reaction. As a catalyst, we initially chose to employ the commercial complex IPrAuNTf_2_ (IPr=N,N’‐(2,6‐diisorpopylphenyl)imidazole‐2‐ylidene, NTf_2_^−^=bis(trifluoromethylsulfonyl)imide), that already proved competent in our previous investigations on the intermolecular alkyne hydroarylation with arenes.[^41^, ^42^, ^43^] Furthermore, the presence in the complex of a weakly coordinated NTf_2_^−^ anion avoids the need for a silver salt as co‐catalyst to remove more strongly bound anionic ligands such as halides.

Prompted by our recent findings on the synergistic role of gold catalysis and ILs in intermolecular hydroarylation reactions,[^42^, ^43^] we addressed our initial efforts towards a screening of ILs as reaction media. Interestingly, through the combined use of NTf_2_^−^ anion and BMIM as countercation, almost quantitative yields of **2 a** could be reached within 30 minutes with 0.5 mol % gold catalyst (Table 1, entry 1). The IL with NTf_2_^−^ as anion provided by far the best results, as in the case of the previously investigated intermolecular hydroarylations.[^41^, ^42^, ^43^] On the other hand, upon changing the IL anion the results were somewhat different from those obtained in the intermolecular variant, in that also BF_4_^−^ yielded a system featuring reasonable catalytic activity (Table 1, entry 4). We also discovered that, in order to avoid reproducibility issues, use of freshly supplied ILs is mandatory whenever the counteranion is a perfluorocomplex, since these counteranions undergo slow decomposition via a likely hydrolytic pathway, and the presence of impurities deriving from such decomposition negatively affects the catalytic performance.^[44]^

**Table 1:** Effect of the ionic liquid on the reaction performance.

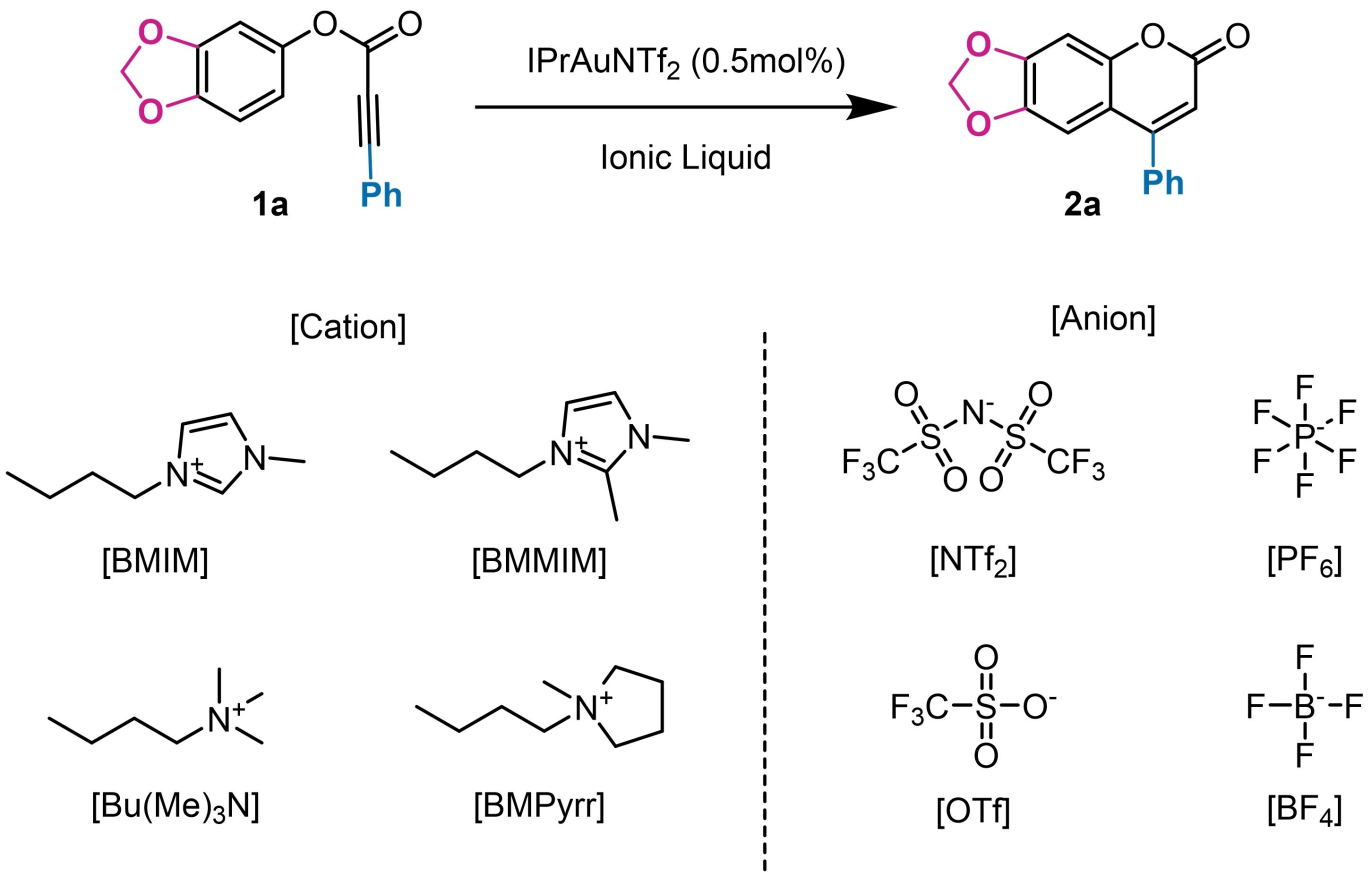					
Entry	[Cation]	[Anion]	T (°C)	Time (h)	Yield (%)^[a]^
1	[BMIM]	[NTf_2_]	40	0.5	97
2	[BMIM]	[OTf]	40	4	NR^[b]^
3	[BMIM]	[PF_6_]	40	4	NR^[b]^
4	[BMIM]	[BF_4_]	40	1 2	52 97
5	[BMMIM]	[BF_4_]	40	6 24	15 48
6	[BMMIM]	[BF_4_]	50	6 24	31 95
7	[BMIM]	[PF_6_]	50	3	NR^[b]^
8	[BMMIM]	[PF_6_]	50	3	NR^[b]^
9	[BMIM]	[OTf]	50	24	NR^[b]^
10	[BMMIM]	[OTf]	50	6 24	38 >99
11	[BMMIM]	[NTf_2_]	40	0.5	>99
12	[Bu(Me)_3_N]	[NTf_2_]	40	0.5 1	93 >99
13	[BMPyrr]	[NTf_2_]	40	1 2	85 >99

[a] Yields were determined by ^1^H NMR spectroscopy with dimethylsulfone or 1,2‐dimethoxyethane as internal standards; [b] NR: no reaction.

Interestingly, the nature of the cation had also some effect on the overall catalytic performance, which was most evident with the anions providing lower activity. Indeed, little differences were observed with NTf_2_^−^ as the anion (entries 11, 12 and 13); only with the cation BMPyrr the activity was significantly lower, though still high (entry 13). On the contrary, use of the BMMIM cation instead of BMIM caused an impressive activation of the catalytic system when OTf^−^ was used as anion (Table 1, entry 10). An opposite trend was observed for BF_4_^−^ (Table 1, entries 4 and 5), whereas the system with PF_6_^−^ as anion remained inactive. We tend to attribute this effect to the fact that in comparison with BMIM the BMMIM cation lacks the capacity to act as a hydrogen bond donor.^[43]^ This capacity may give rise to strong interionic interactions, when the cation is coupled with anions exhibiting strong hydrogen bond basicity, with the consequent formation of microheterogeneities in the ionic liquid phase.^[45]^ In the absence of such strong interionic interactions, (*i. e*. when the anion has low hydrogen bond basicity, as in the case of NTf_2_^−^), the effect of the cation vanishes and can present itself again only when the change in the physico‐chemical properties is so large to alter the solubility of the substrate or of the catalyst in the ionic liquid.

Once the optimal ionic liquid has been selected, we turned our attention to the minimization of the gold content in order to obtain efficient catalysis (Table 2).

**Table 2:** Effect of acid addition on the reaction performance.

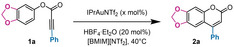				
Entry	[Au] (mol %)	HBF_4_ (mol %)	Time (h)	Yield (%)^[a]^
1	0.5	‐	0.5	97
2	0.5	20	0.5	>99
3	0.1	‐	24	traces
4	0.1	20	1 1.5	79 98
5	0.05	20	1 3	30 93
6	0.01	20	3 5 24	35 61 >99
7	‐	20	24	NR^[b]^

[a] Yields were determined by ^1^H NMR spectroscopy with dimethylsulfone or 1,2‐dimethoxyethane as internal standards; [b] NR: no reaction.

As mentioned above, using our system it was possible to lower the gold content down to 0.5 mol % without compromising the catalytic performance. Moreover, by adding 20 mol % of a Brønsted acid (*i. e*. HBF_4_⋅Et_2_O) as co‐catalyst, it was possible to further lower the gold content to 0.01 mol % without significant erosion of the chemical outcome (yield=99 %; Table 2, entry 6).

**Scheme 2:**
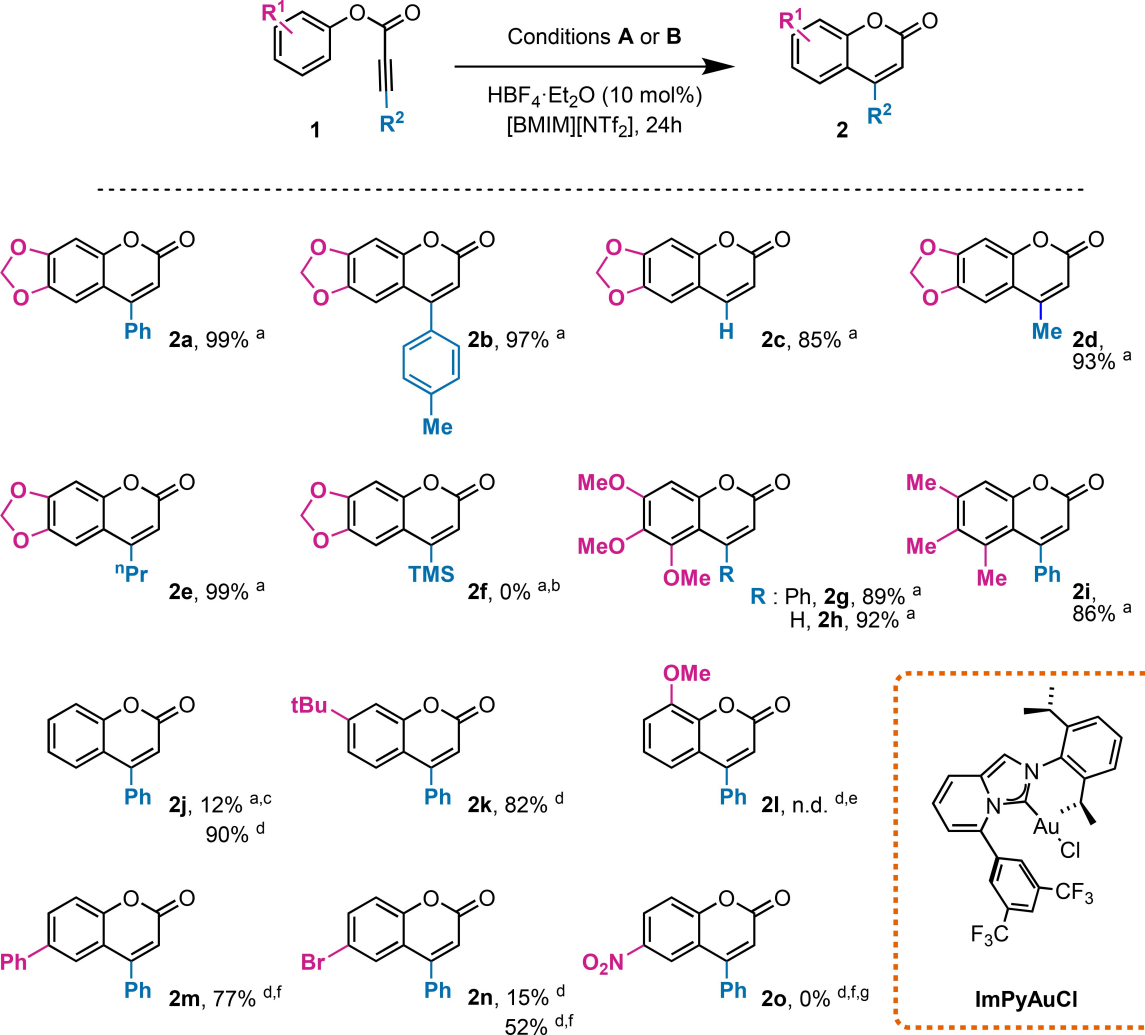
Reaction scope (isolated yields are reported. ^a^ Conditions **A**: [**1**]=0.67 M, 0.05 mol % IPrAuNTf_2_ at 40 °C; ^b^ No reaction observed; ^c^ 84 % of **1 j** was recovered; ^d^ Conditions **B**: [**1**]=0.67 M, 0.5 mol % IPrAuNTf_2_ at 80 °C; ^e^ **2 l** was detected by ^1^H NMR spectroscopy, however purification by column chromatography was impractical due to the presence of multiple by‐products; ^f^ The complex ImPyAuCl was employed instead of IPrAuNTf_2_ with 1 equiv. AgSbF_6_ as activator; ^g^ extensive hydrolysis of **1 o** was detected by ^1^H NMR spectroscopy.

The possible roles of the acid additive are to assist protonolysis, which may be the rate determining step of the reaction, and to keep the catalyst active when lower amounts of gold(I) complex are employed.^[37]^ In the latter context, the acid cocatalyst is supposed to restore the active monomeric gold complex by attacking off‐cycle intermediates such as *gem*‐diaurated alkynyl species.[^46^, ^47^] The effect of the acid additive is evident when 0.1 mol % of gold(I) complex was employed: the system is completely static/poisoned under neutral conditions (Table 2, entry 3). On the other hand, as soon as HBF_4_⋅Et_2_O is added the complete restoration of the catalytic activity occurs (Table 2, entry 4). We could push the reaction conditions until reducing the amount of IPrAuNTf_2_ to 0.01 mol % and still a quantitative yield was obtained after 1 day reaction (Table 2, entry 6). To our knowledge, it is the first time that gold(I)‐catalysts were employed in such low amounts over the framework of C−H bond functionalization reactions. At this point, a sample of 15 substrates was taken into consideration to test the synthetic application and the generality of the method (Scheme 2).

Maintaining the acid additive load at 10 mol % and the reaction time at 24 h we could still obtain quantitative conversion of **1 a** to yield the corresponding coumarin **2 a**, employing reduced amount of the commercial gold complex IPrAuNTf_2_ (conditions **A**). Installing a more donating group at the alkyne moiety, such as *p*‐tolyl (**1 b**) or alkyl chains with different lengths (**1 d** or **1 e**) showed a low influence on the catalytic performance allowing to achieve quantitative conversion of the alkynoates and isolated yields above 90 %. On the other hand, an even more electron‐rich alkyne, such as **1 f**, resulted completely inactive toward the employed conditions.

The effect of substituents at the *meta* positions of the phenolate ring was also tested. 3,4,5‐Trisubstituted‐phenol derivatives **1 g** and **1 i** were employed achieving good yields. As well, comparable efficiencies are found when a terminal alkyne moiety is employed (**1 c** and **1 h**).

Unfortunately, poor yields were obtained with substrate **1 j** bearing an unsubstituted phenolate ring, (yield=12 %), however, by increasing the amount of catalyst (0.5 mol %) and reaction temperature (80 °C, conditions B), **2 j** was conveniently isolated in 90 % yield. Substrate **1 k** was well adapted to the scope, with 82 % isolated yield and complete selectivity toward the reported isomer **2 k**. Surprisingly, moving to substrate **1 l** we encountered unexpected selectivity issues which made unpracticable the purification of **2 l** by column chromatography.

Finally, we tackled the suitability of more deactivated substrates bearing different electron withdrawing groups at the *para* position of the aryl moiety. A first attempt was conducted employing conditions **B** and **1 n** as substrate. Here, only 15 % yield was achieved after 24 h. Ligand design provided a solution to such a limited catalyst performance, by tuning the electronic properties of the gold metal center with a Buchwald‐type pendant installed on the pyridine‐derived NHC ligand (ImPy). In fact, some of us have recently reported on the beneficial effect of CF_3_‐containing ligands on electrophilic gold‐catalyzed processes.[^48^, ^49^] Satisfyingly, an impressive enhancement of the catalytic performance was detected, achieving **2 n** in 52 % yield. Activation of the 4‐phenyl derivative **1 m** was as well accomplished, achieving 77 % yield of **2 m**, while the more deactivated nitro compound **1 o** showed no conversion to hydroarylation products. Analysis of the reaction crude by ^1^H NMR spectroscopy consistently showed hydrolysis of the ester group as the only reaction taking place, with a rate increasing with higher gold complex loading: neither **1 o** nor **2 o** were detected after one day of reaction when 2 mol % ImPyAuCl was employed.

## Conclusions

A new ionic liquid‐based system was reported, showing improved catalytic performances in the gold‐catalyzed intramolecular hydroarylation of aryl alkynoates to yield coumarins. Extremely low catalyst loadings could be employed in case of activated substrates with a good tolerance toward the alkyne substituent, allowing to access a broad variety of 4‐susbtituted coumarins. Differently substituted phenolate rings could also be incorporated in the reaction scope using harsher conditions and a higher amount of gold catalyst, although still lower than what is generally employed in the literature in the context of alkyne hydroarylation processes. Finally, tuning the electronic properties of the metal center by means of the reported ImPy‐NHC ligand, we could access coumarins **2 m** and **2 n** with analogous results to the ones obtained by Shi and He with 5 mol % AuCl_3_/3AgOTf catalyst,^[23]^ which have been up to now the best result reported in gold catalysis for those substrates. Further optimization and extension of this catalytic system to other substrate classes is currently underway.

## Conflict of Interests

The authors declare no conflict of interest.

1

## Supporting information

As a service to our authors and readers, this journal provides supporting information supplied by the authors. Such materials are peer reviewed and may be re‐organized for online delivery, but are not copy‐edited or typeset. Technical support issues arising from supporting information (other than missing files) should be addressed to the authors.

Supporting Information

Supporting Information

## Data Availability

The data that support the findings of this study are available in the supplementary material of this article.
