# Supplementary material for: An Improved Gold(I) Catalytic System for the Preparation of Coumarins via Intramolecular Cyclization
Source: Chem Asian J. 2024 Nov 9;20(1):e202400725. doi: 10.1002/asia.202400725 (PMC11697228; doi:10.1002/asia.202400725)
Supplement: Supplementary file 1 — Supporting Information [file ASIA-20-e202400725-s002.pdf]

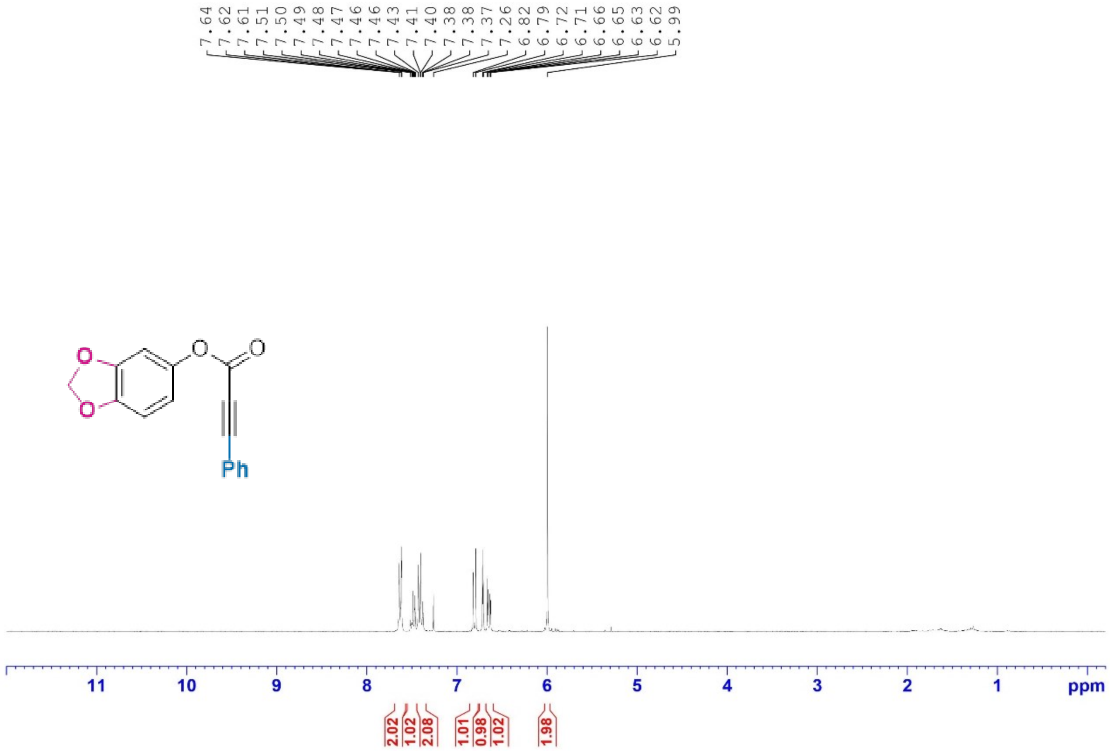

^{1}H NMR (300 MHz, $CDCl_3$) spectrum of **1a**

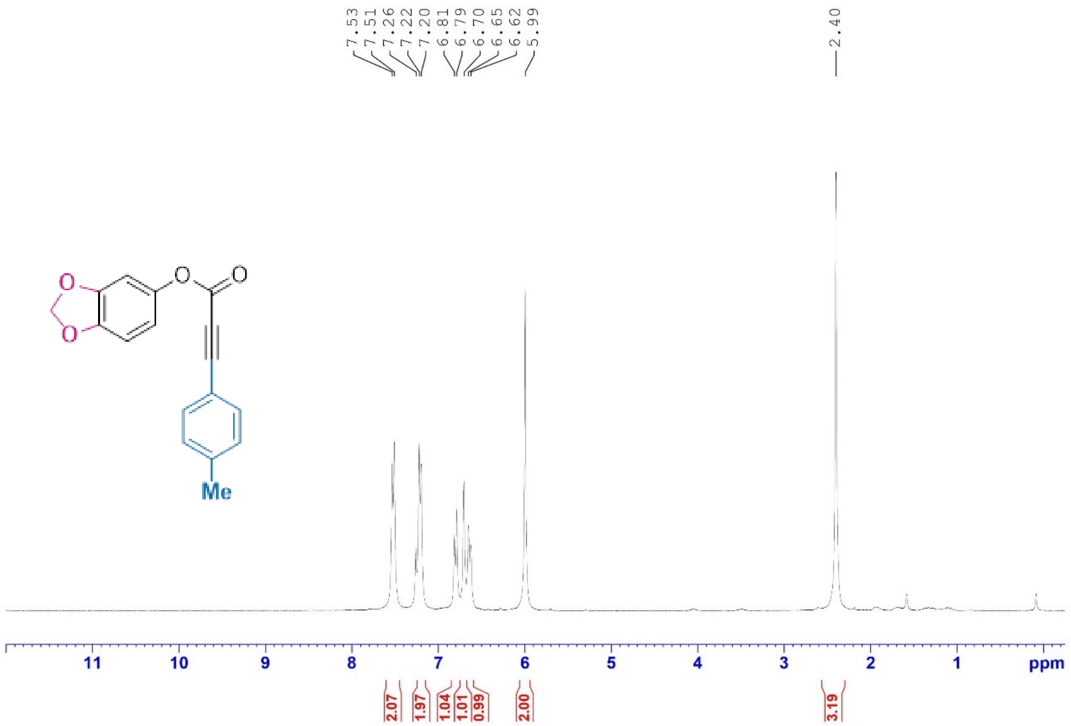

^{1}H NMR (300 MHz, $CDCl_3$) spectrum of **1b**

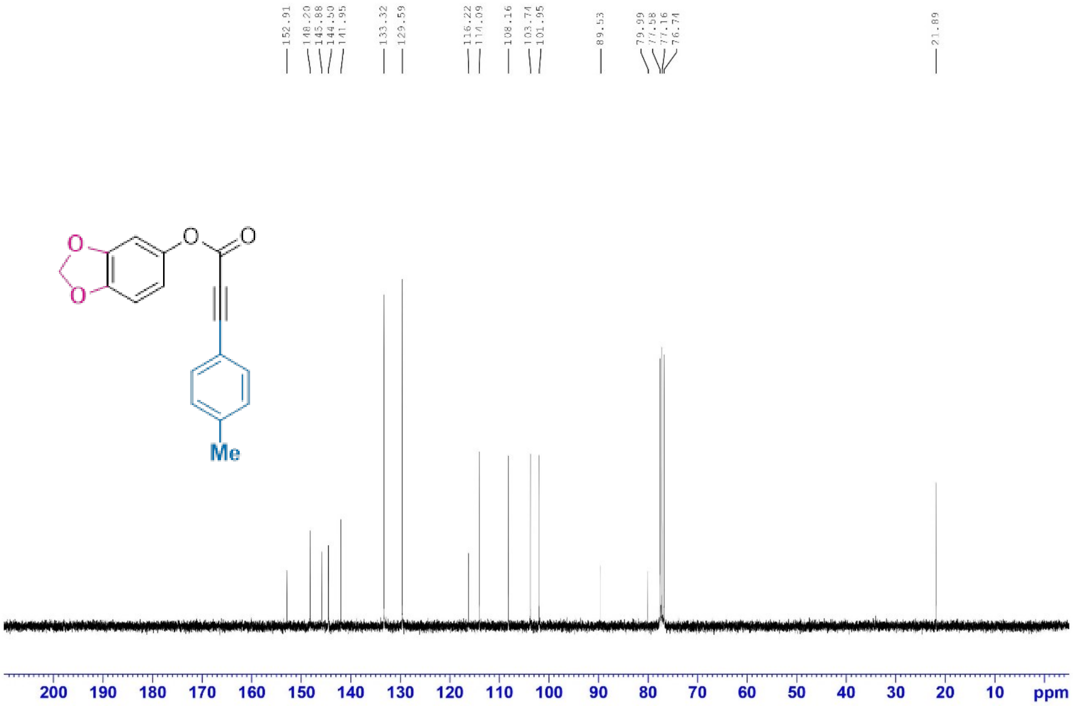

$^{13}C\{^{1}H\}$ NMR (77.5 MHz, $CDCl_3$) spectrum of **1b**

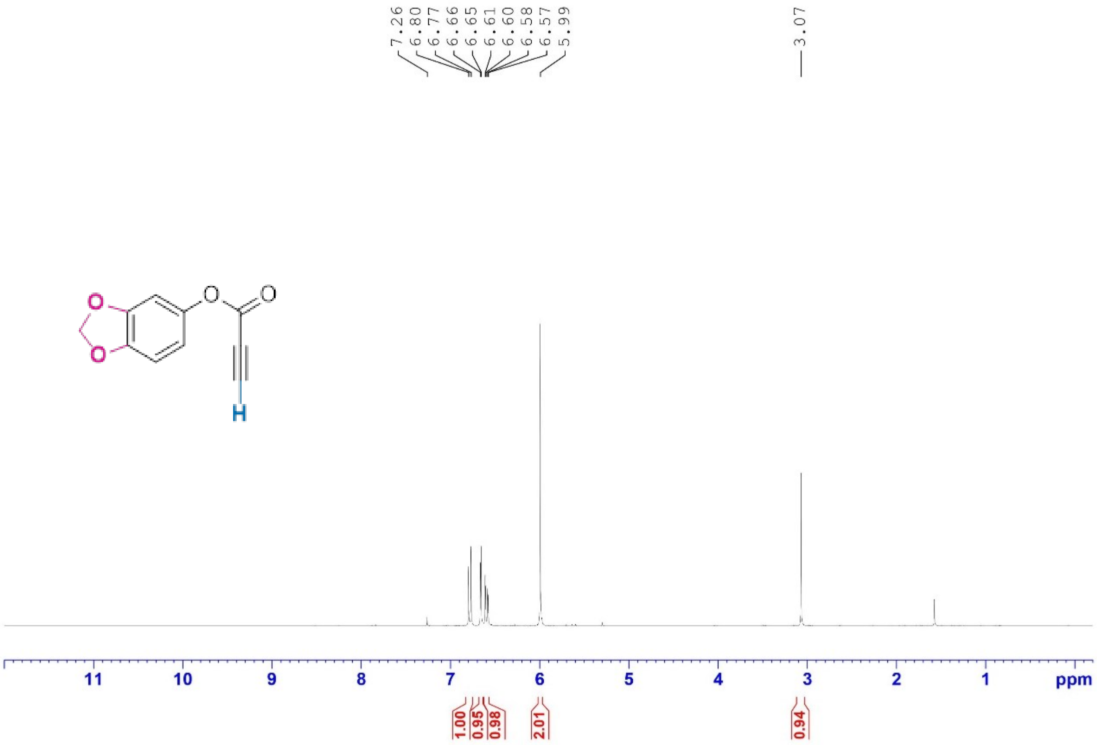

^{1}H NMR (300 MHz, $CDCl_3$) spectrum of **1c**

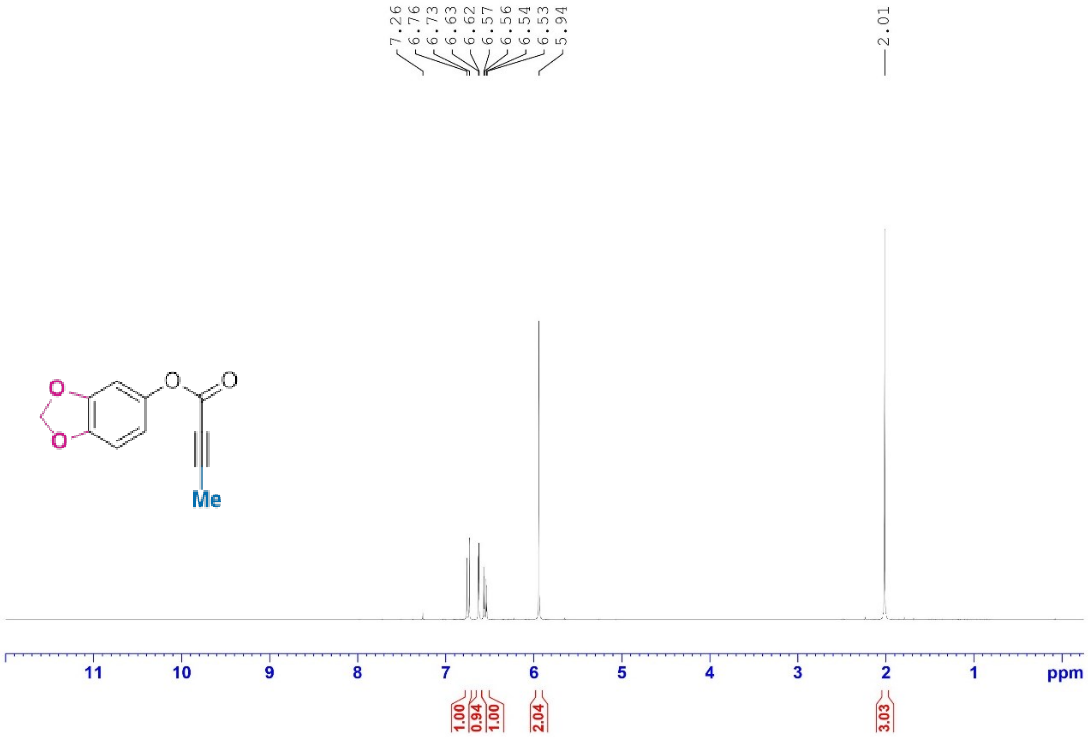

^{1}H NMR (300 MHz, CDCl$_3$) spectrum of **1d**

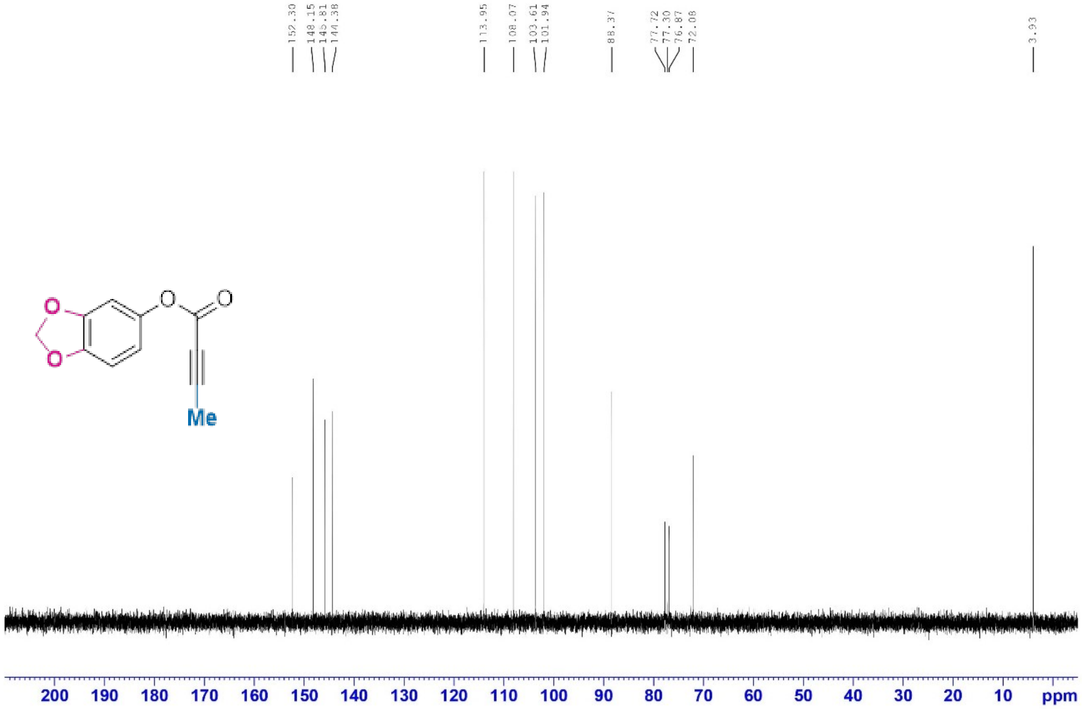

^{13}C{^{1}H} NMR (77.5 MHz, CDCl$_3$) spectrum of **1d**

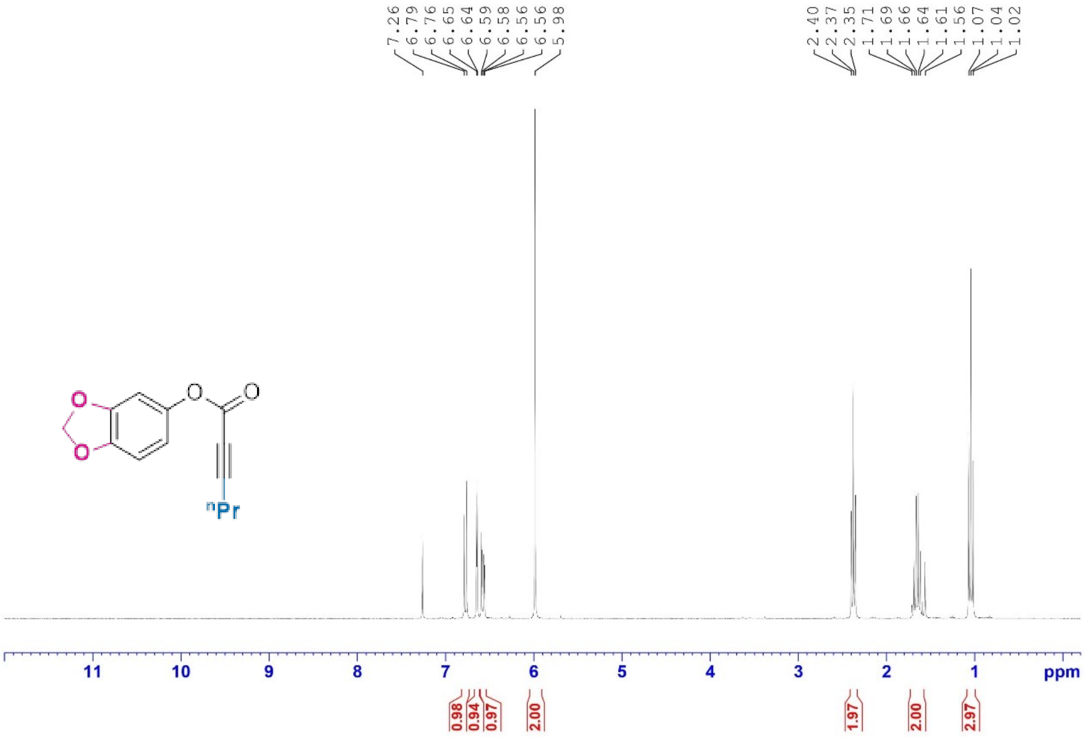

^{1}H NMR (300 MHz, CDCl$_3$) spectrum of **1e**

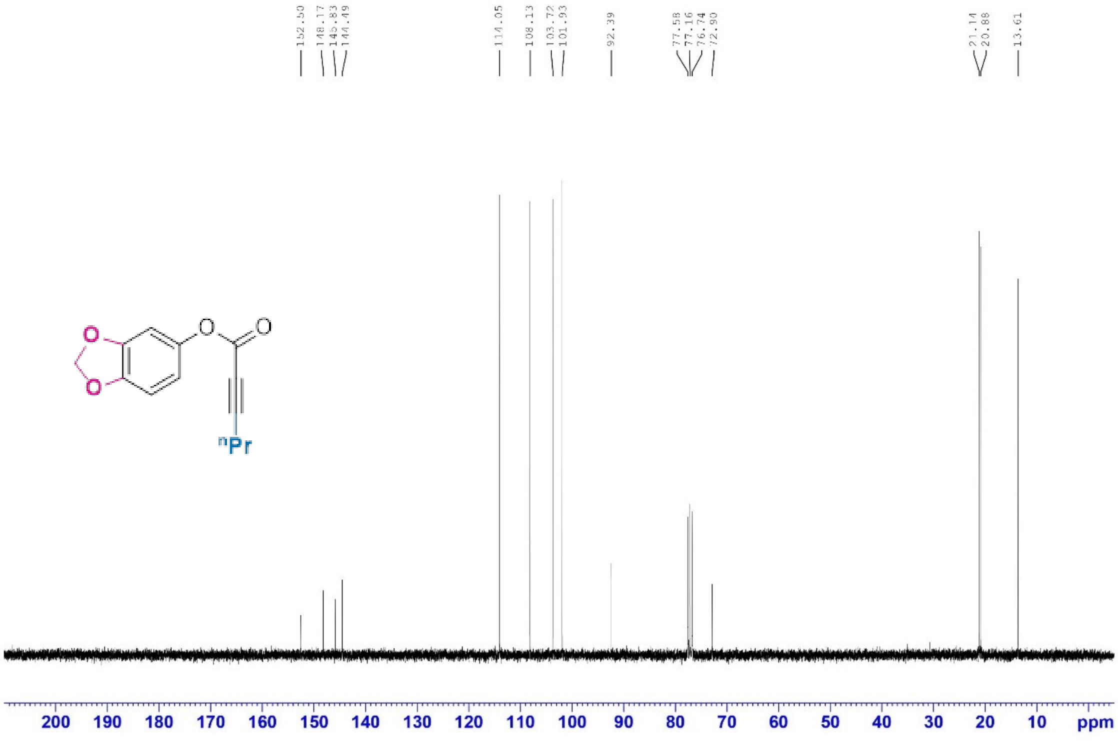

^{13}C{^{1}H} NMR (77.5 MHz, CDCl$_3$) spectrum of **1e**

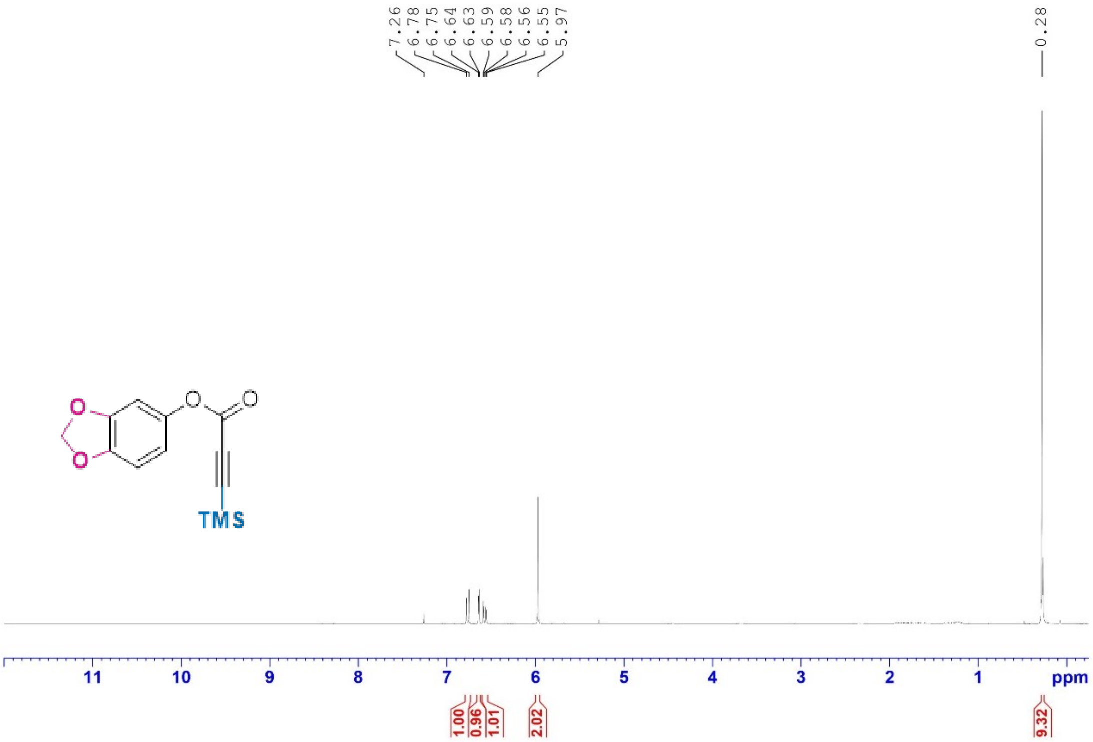

^{1}H NMR (300 MHz, CDCl$_3$) spectrum of **1f**

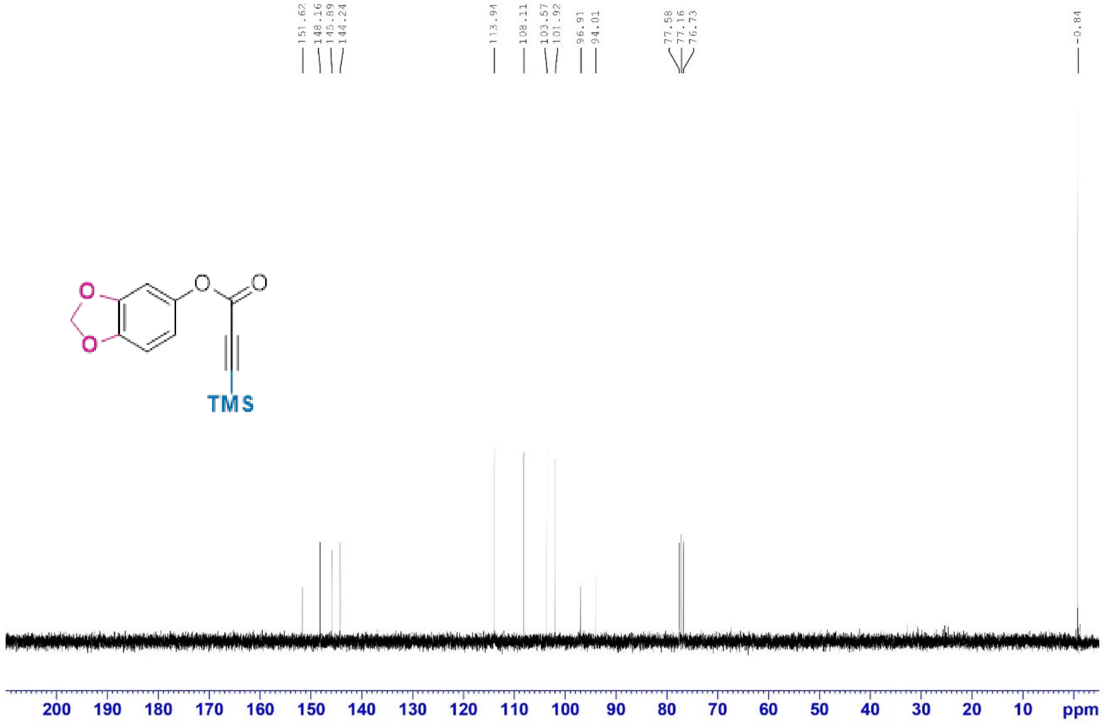

^{13}C{^{1}H} NMR (77.5 MHz, CDCl$_3$) spectrum of **1f**

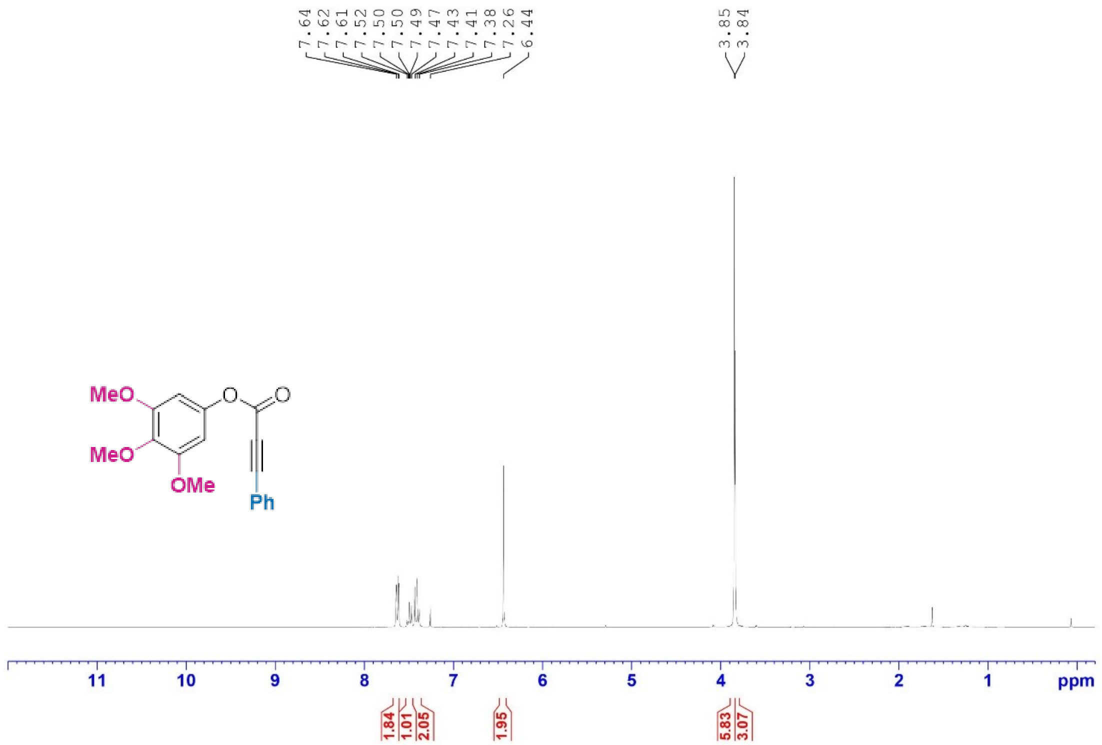

^{1}H NMR (300 MHz, CDCl$_3$) spectrum of **1g**

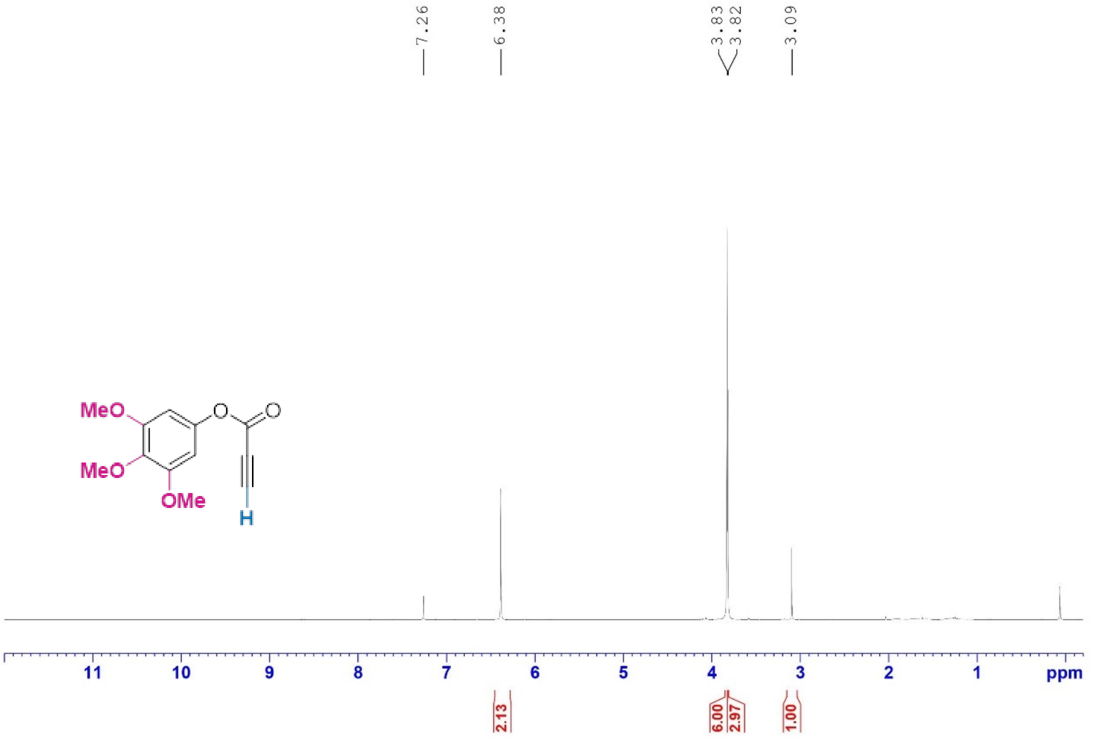

^{1}H NMR (300 MHz, CDCl$_3$) spectrum of **1h**

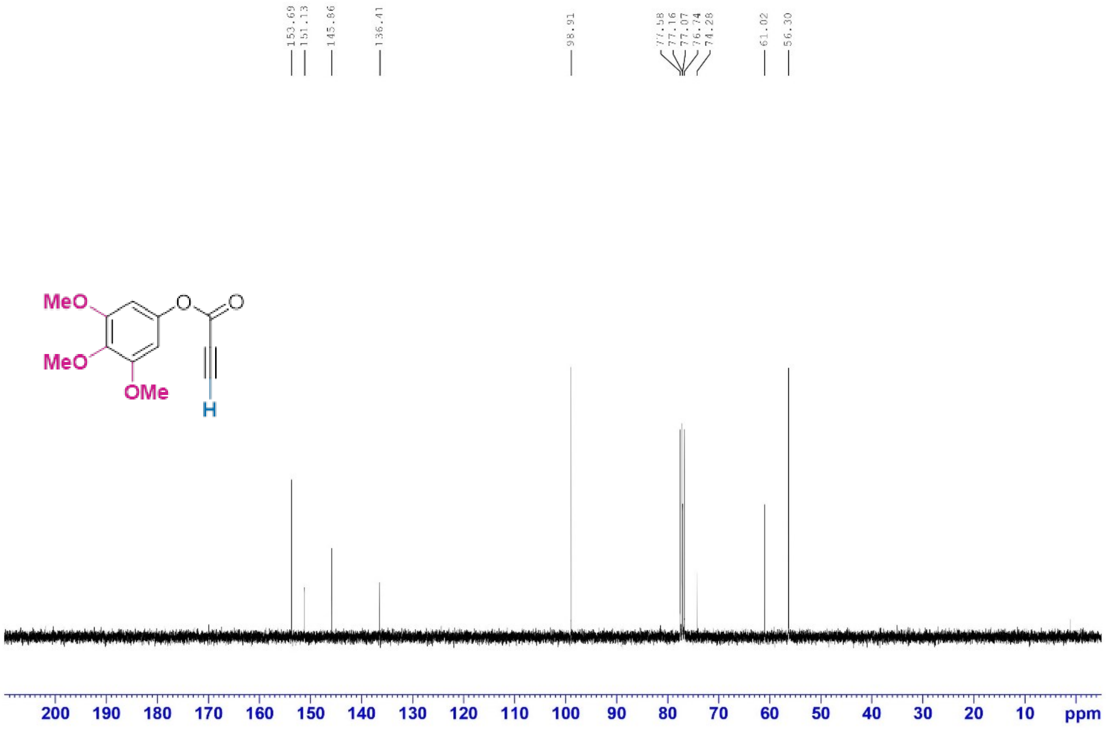

$^{13}C\{^{1}H\}$ NMR (77.5 MHz, $CDCl_3$) spectrum of **1h**

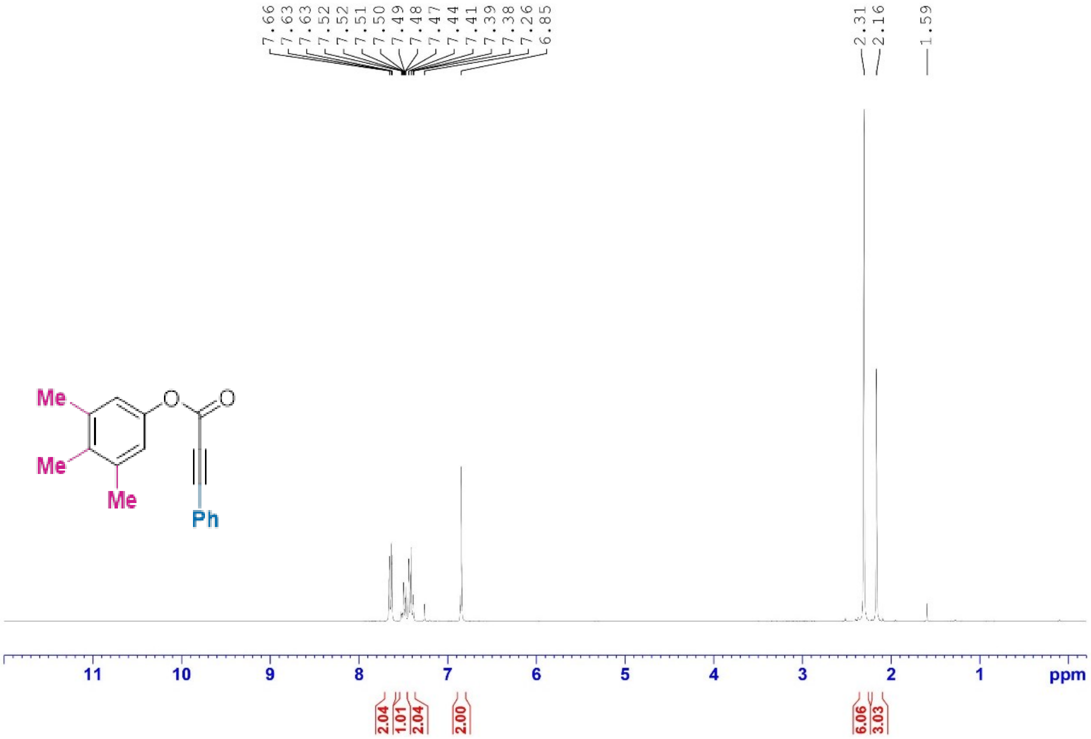

^{1}H NMR (300 MHz, $CDCl_3$) spectrum of **1i**

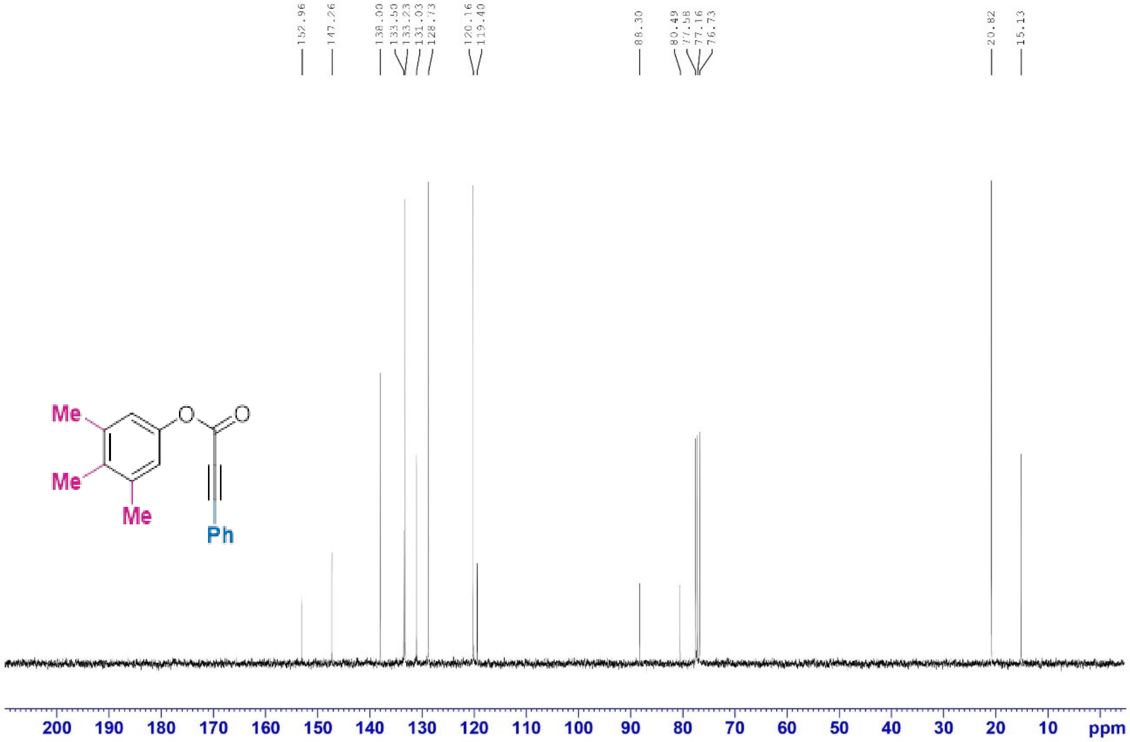

$^{13}C\{^{1}H\}$ NMR (77.5 MHz, $CDCl_3$) spectrum of **1i**

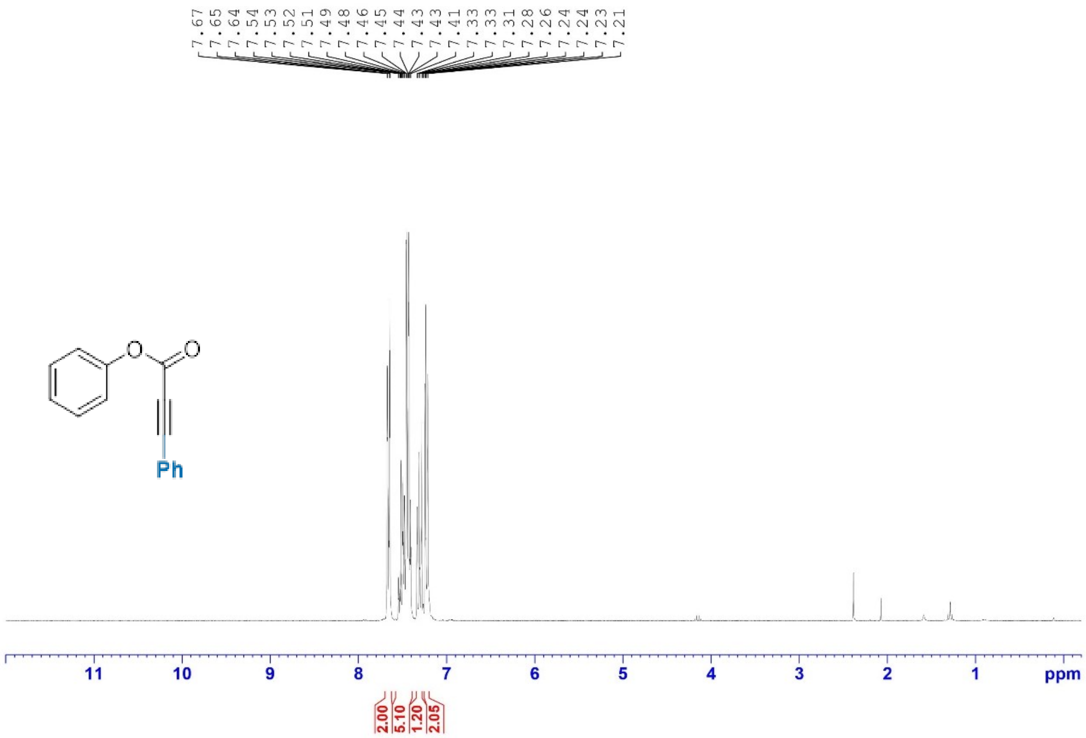

^{1}H NMR (300 MHz, $CDCl_3$) spectrum of **1j**

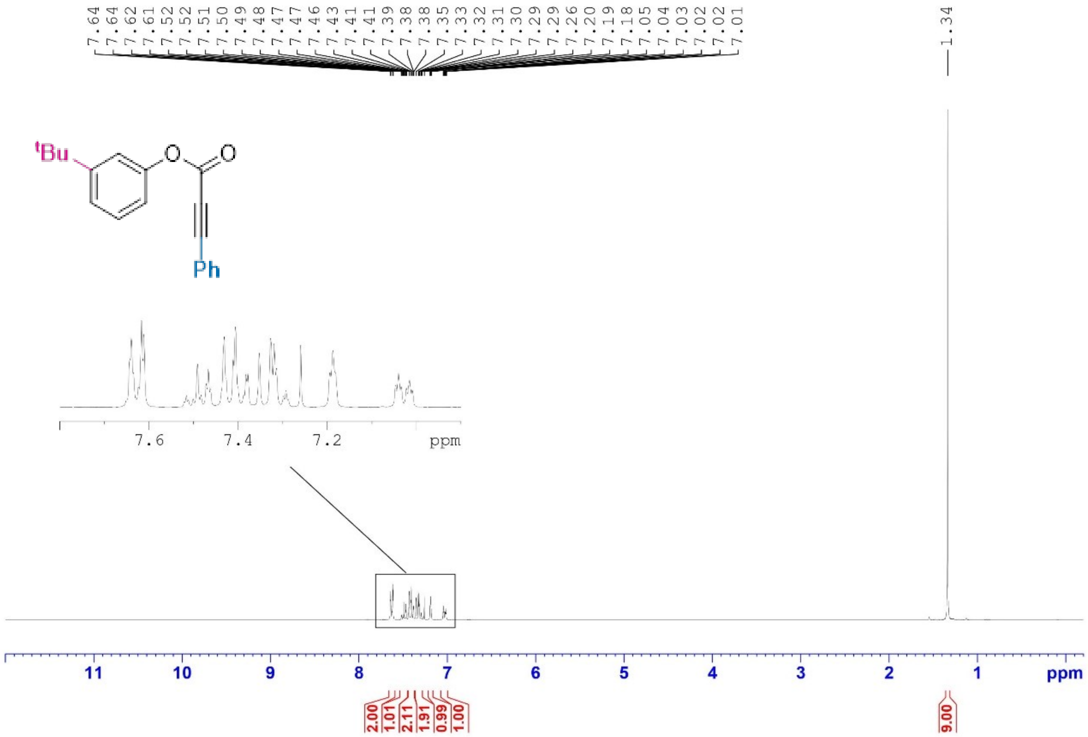

^{1}H NMR (300 MHz, CDCl$_3$) spectrum of **1k**

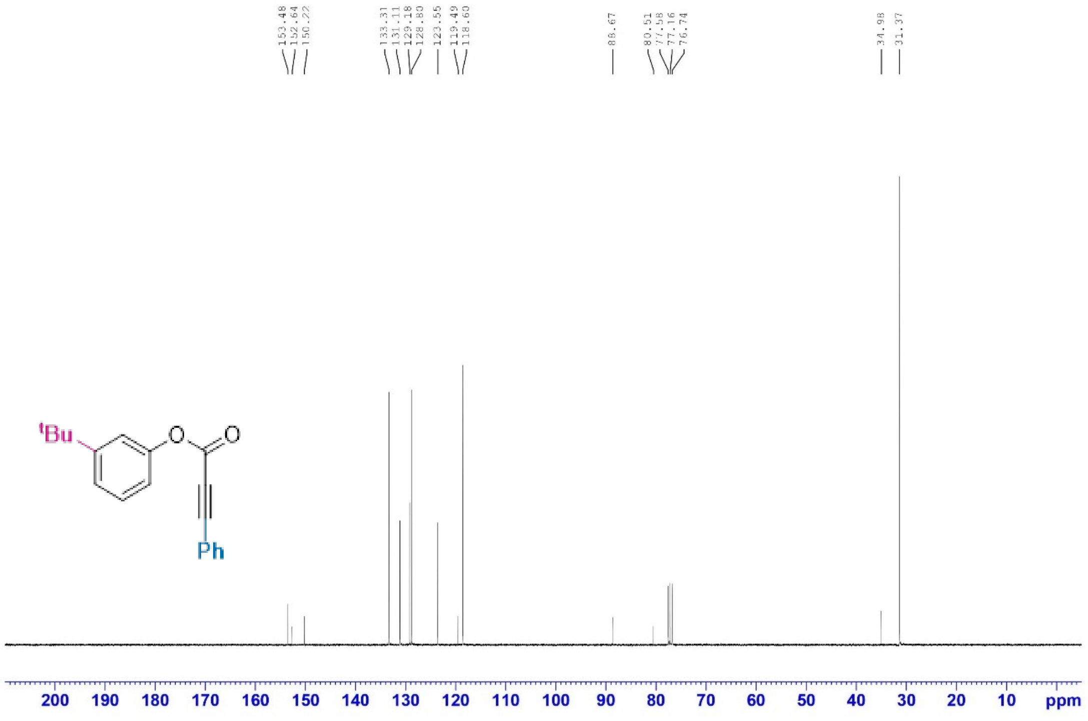

^{13}C{^{1}H} NMR (77.5 MHz, CDCl$_3$) spectrum of **1k**

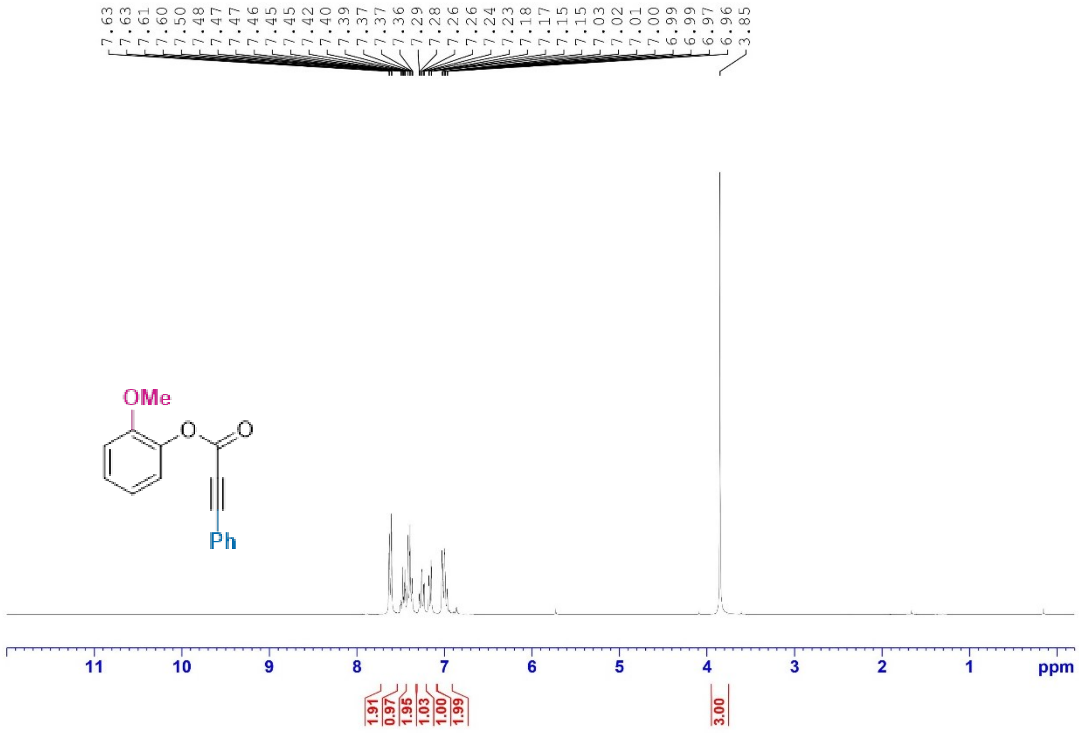

^{1}H NMR (300 MHz, CDCl$_3$) spectrum of **1l**

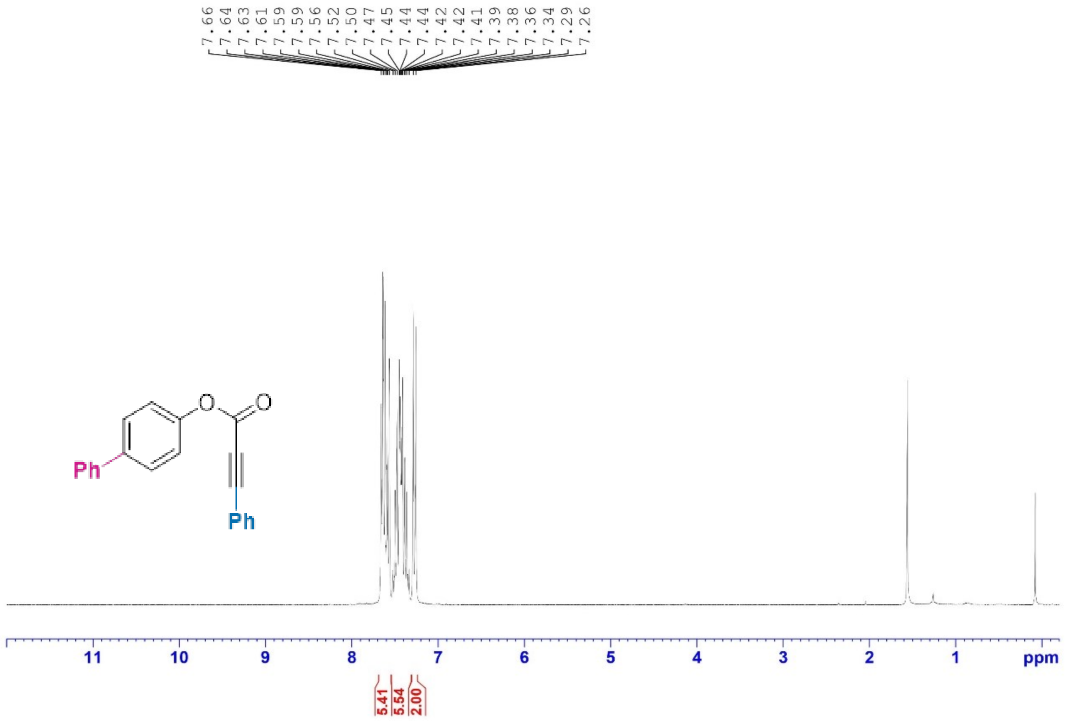

^{1}H NMR (300 MHz, CDCl$_3$) spectrum of **1m**

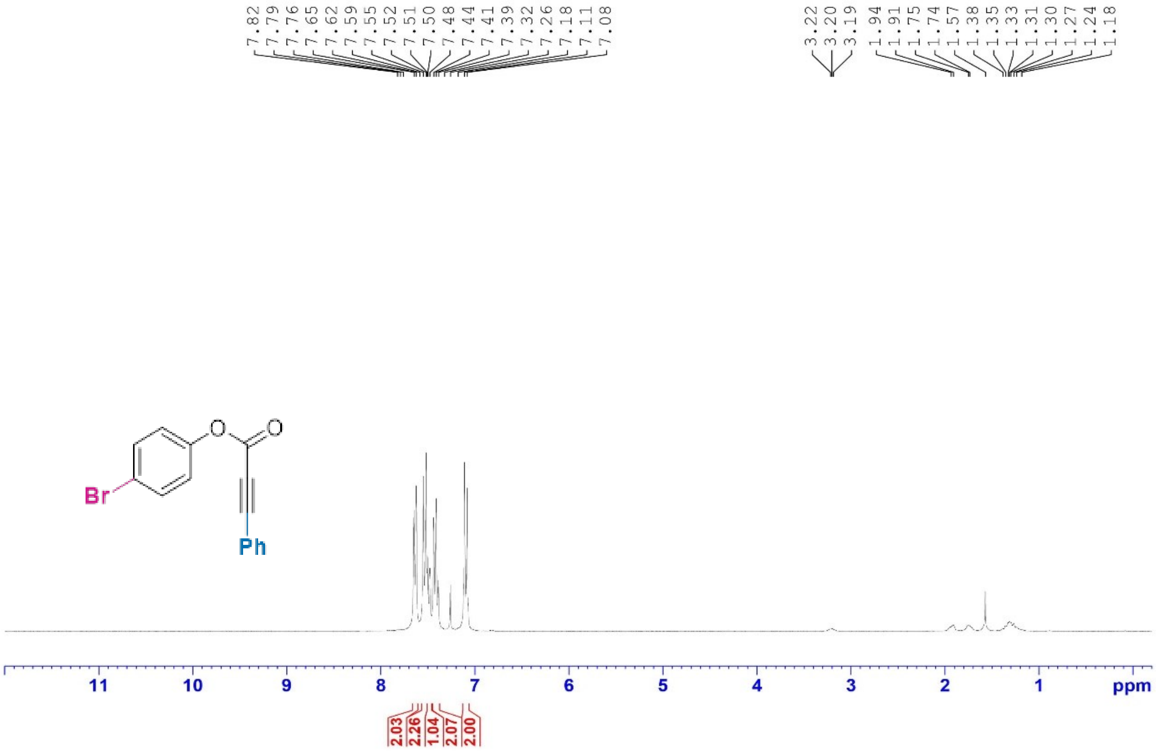

^{1}H NMR (300 MHz, CDCl$_3$) spectrum of **1n**

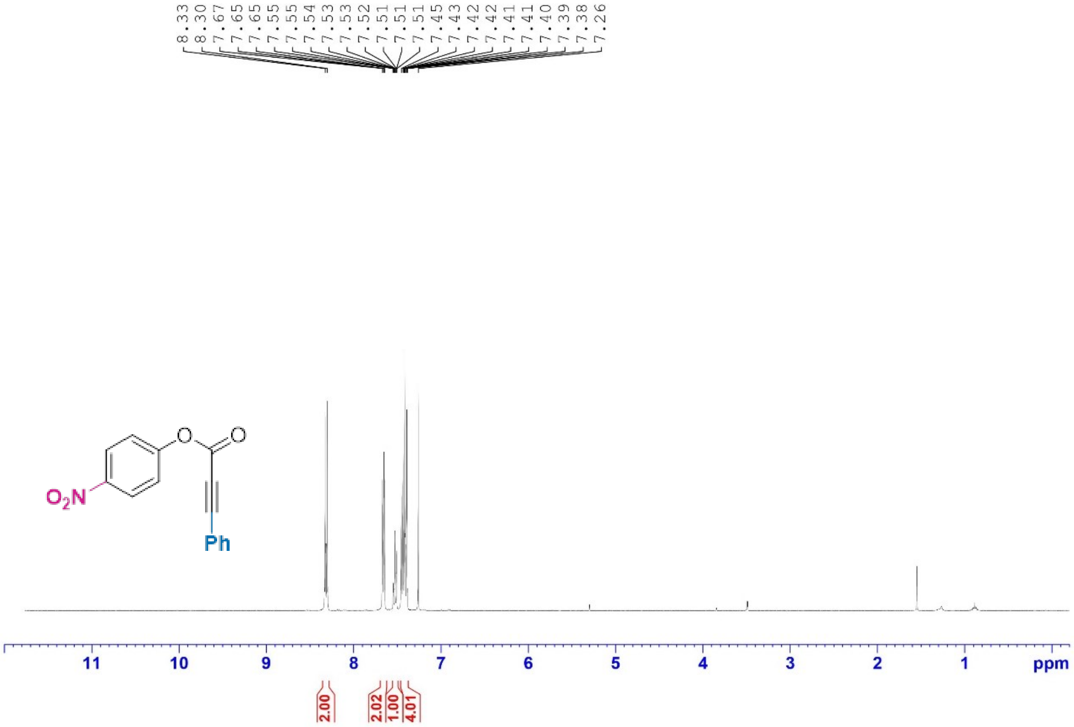

^{1}H NMR (400 MHz, CDCl$_3$) spectrum of **1o**

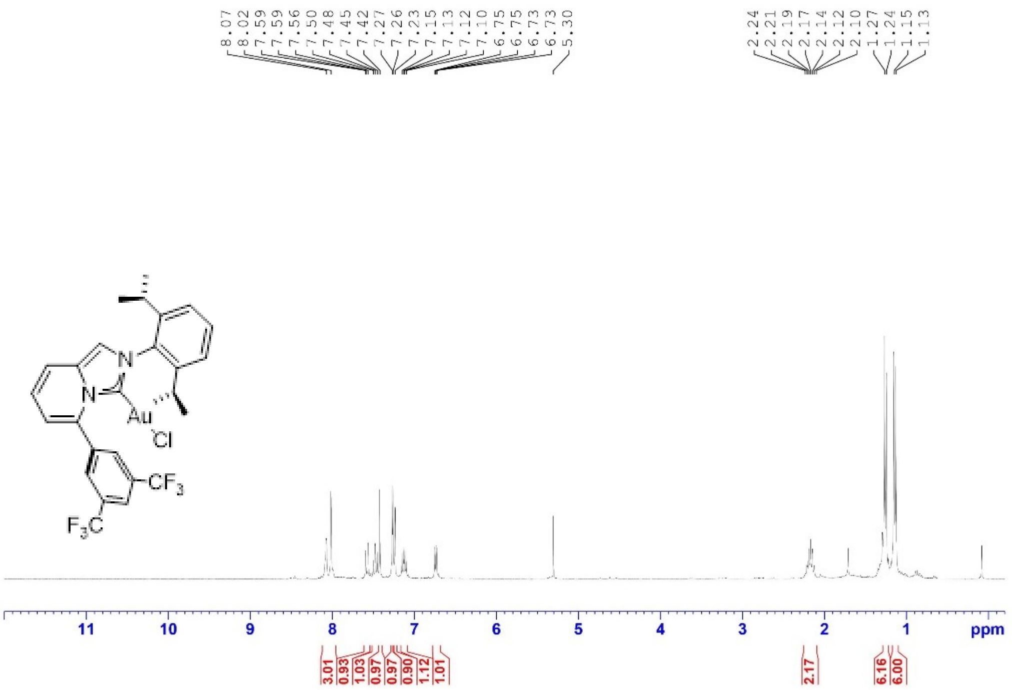

^{1}H NMR (300 MHz, CDCl$_3$) spectrum of **ImPyAuCl**

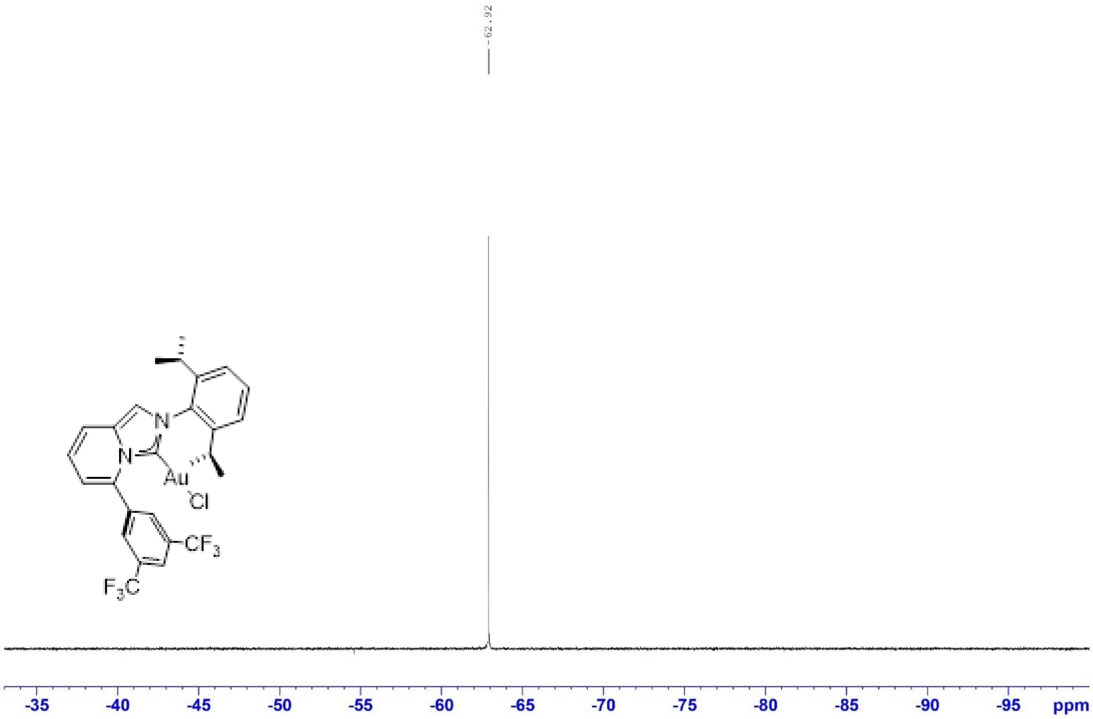

^{19}F NMR (188 MHz, CDCl$_3$) spectrum of **ImPyAuCl**

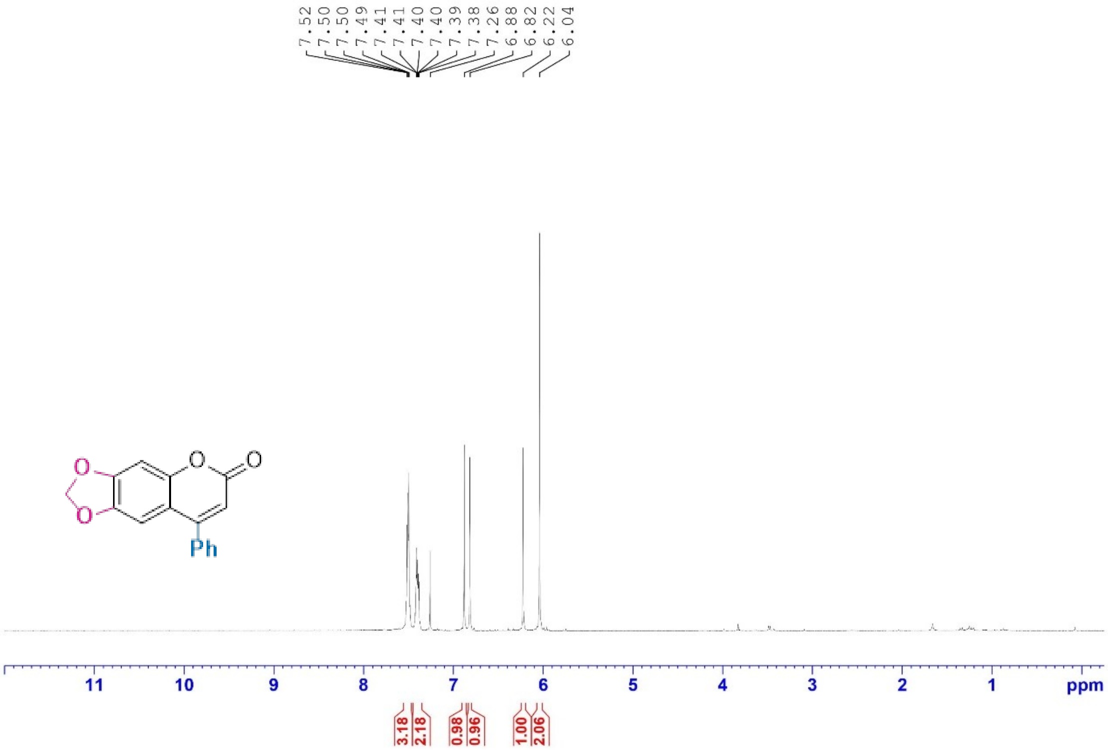

^{1}H NMR (300 MHz, $CDCl_3$) spectrum of **2a**

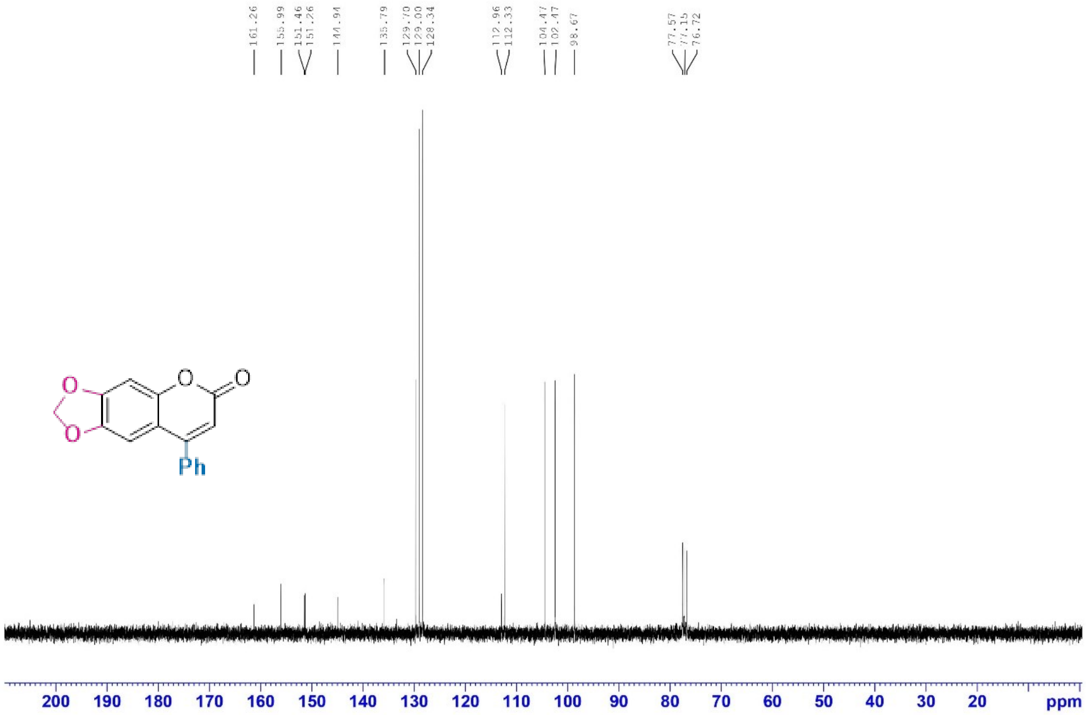

^{13}C{^{1}H} NMR (77.5 MHz, $CDCl_3$) spectrum of **2a**

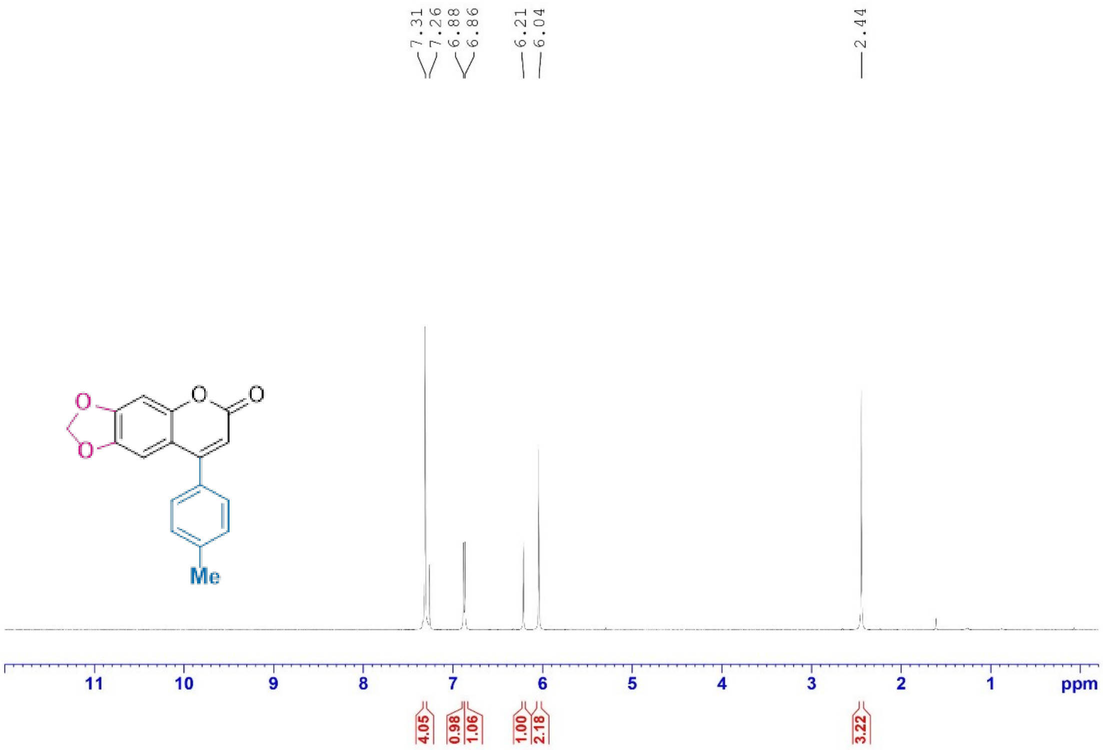

^{1}H NMR (300 MHz, $CDCl_3$) spectrum of **2b**

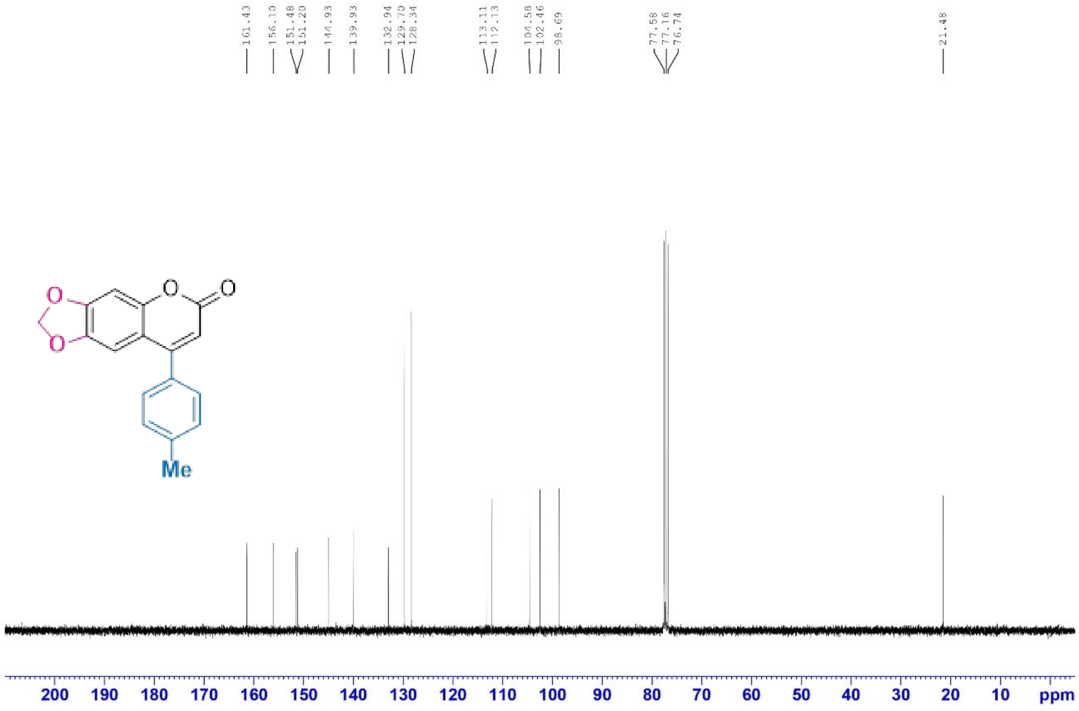

$^{13}C\{^1H\}$ NMR (77.5 MHz, $CDCl_3$) spectrum of **2b**

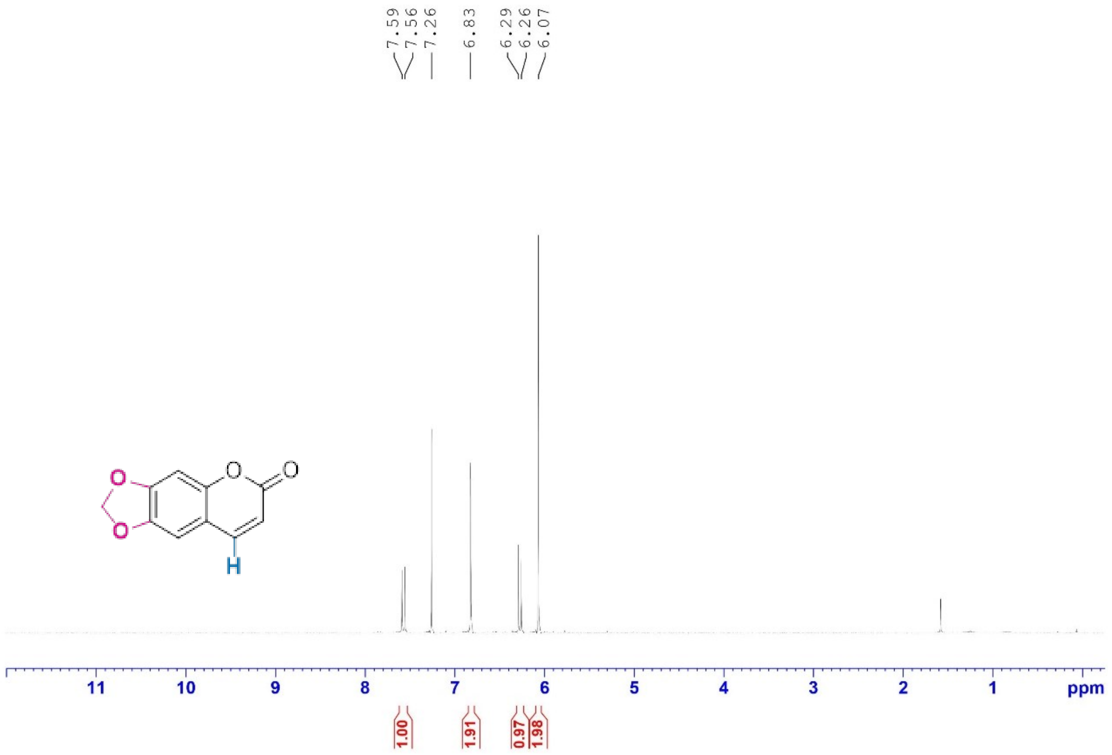

^{1}H NMR (300 MHz, $CDCl_3$) spectrum of **2c**

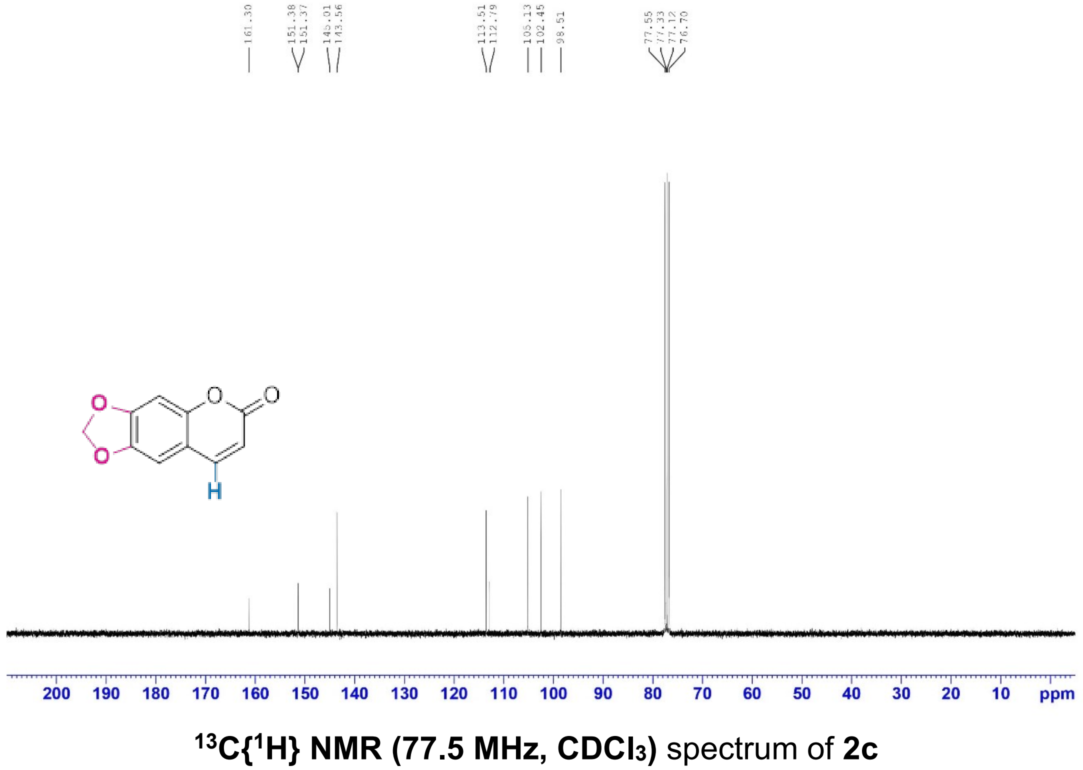

$^{13}C\{^{1}H\}$ NMR (77.5 MHz, $CDCl_3$) spectrum of **2c**

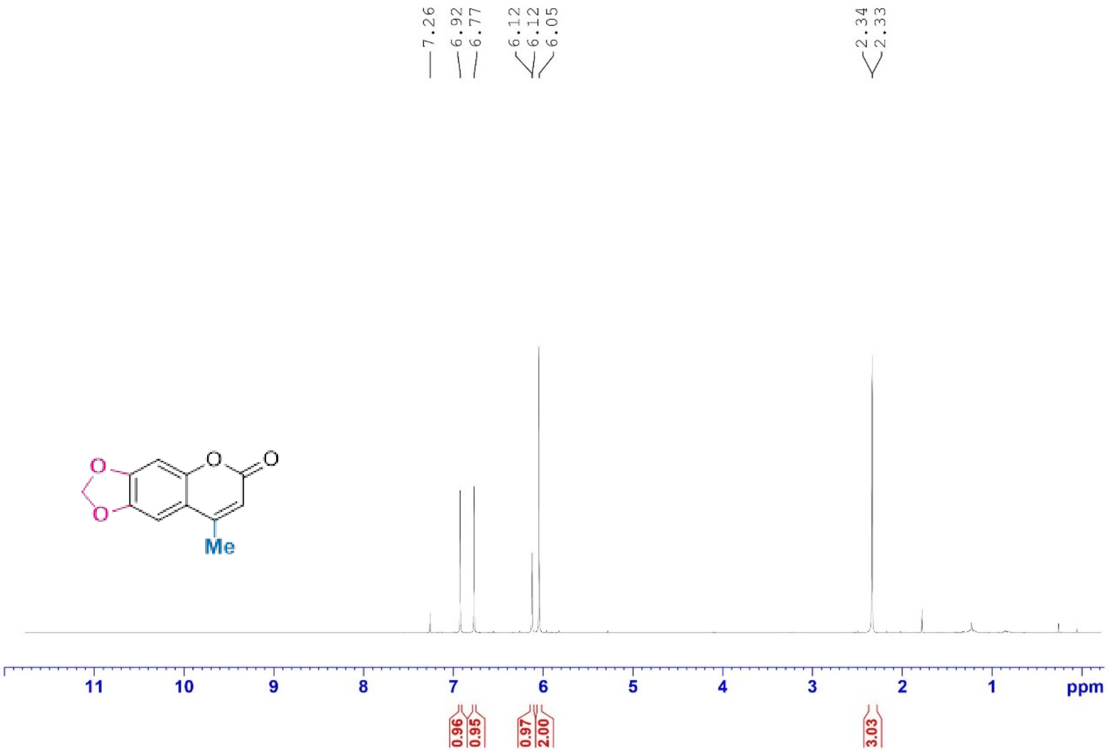

^{1}H **NMR (400 MHz, $CDCl_3$)** spectrum of **2d**

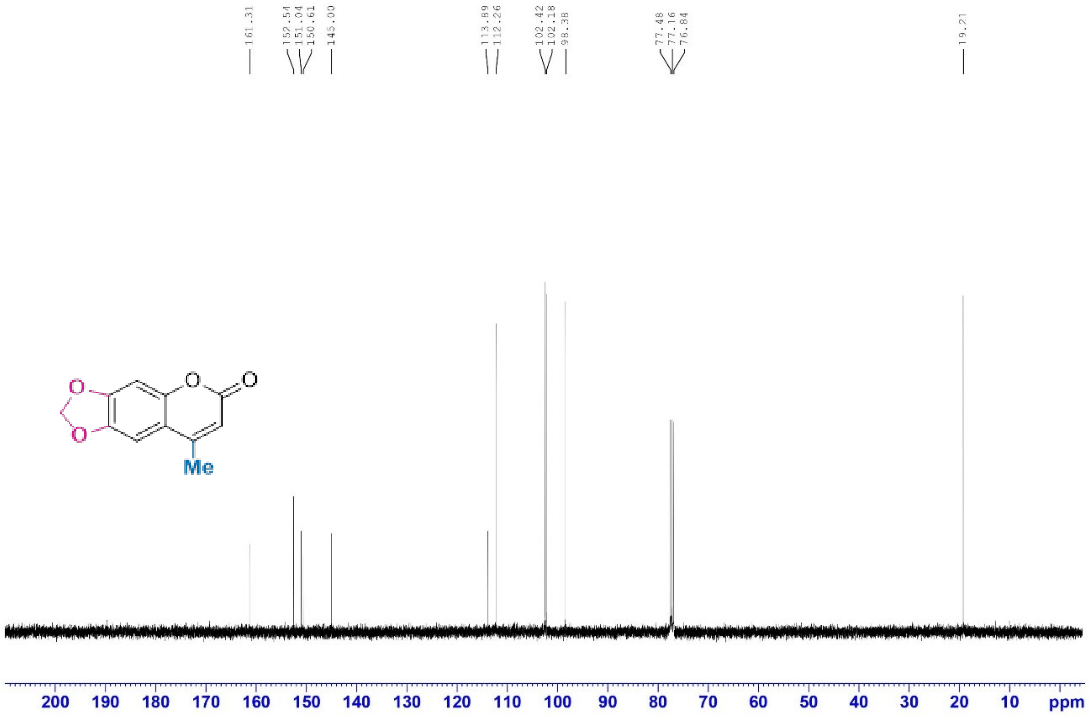

$^{13}C\{^{1}H\}$ **NMR (100.6 MHz, $CDCl_3$)** spectrum of **2d**

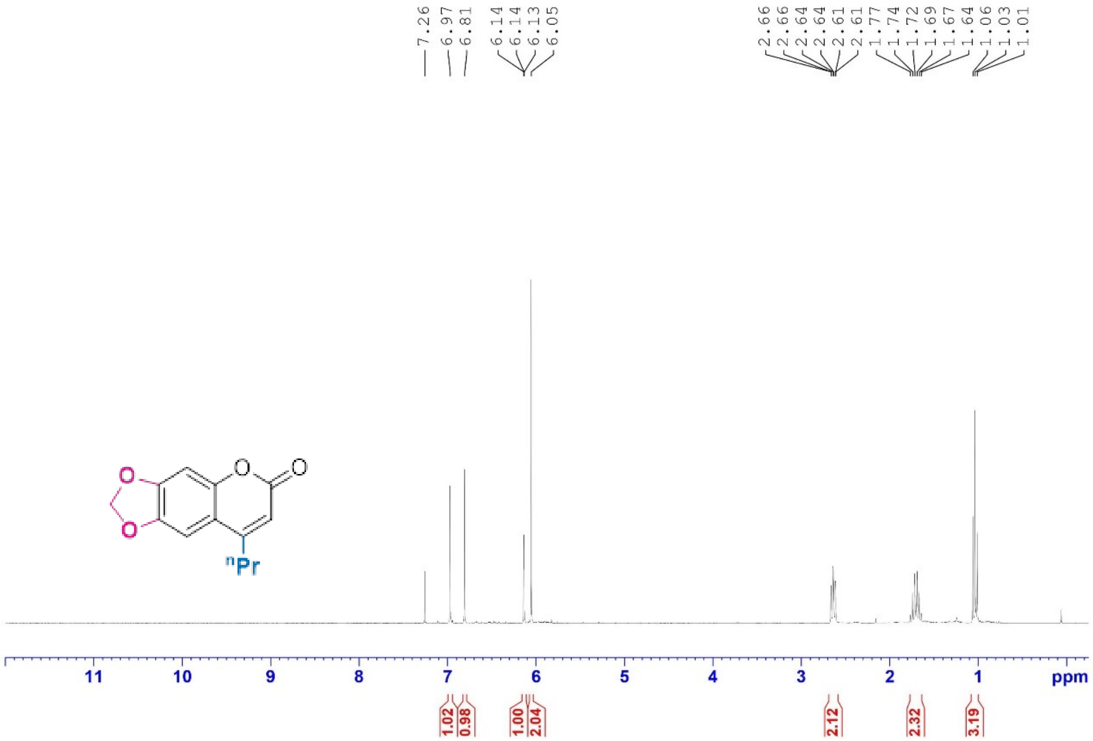

^{1}H NMR (300 MHz, $CDCl_3$) spectrum of **2e**

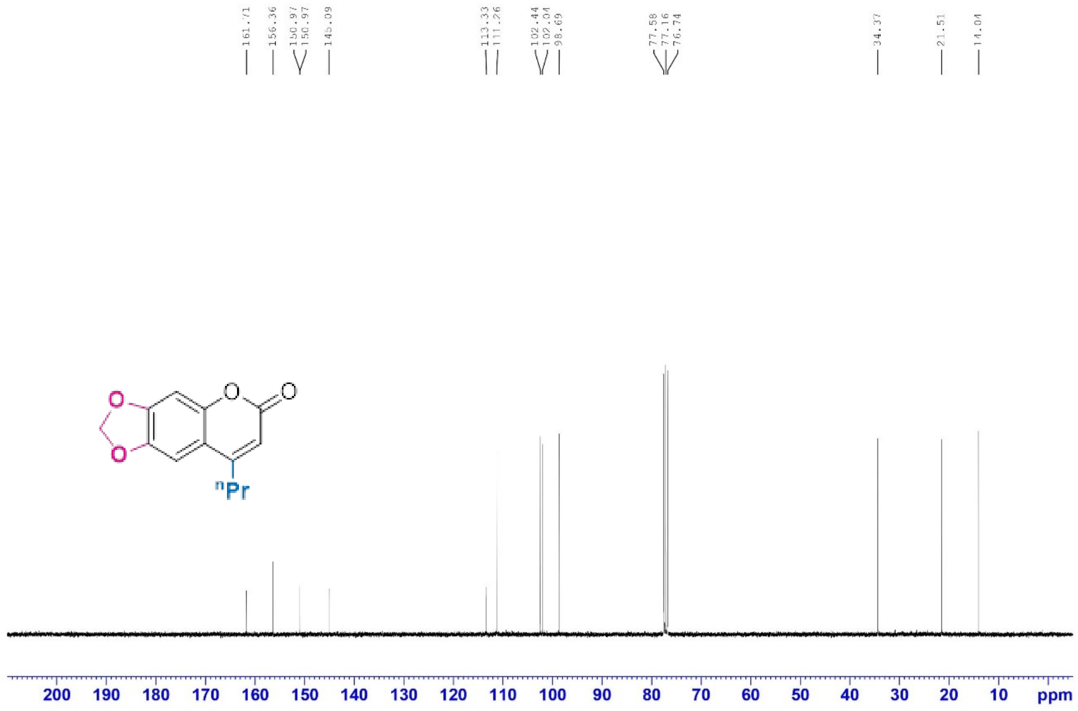

$^{13}C\{^{1}H\}$ NMR (77.5 MHz, $CDCl_3$) spectrum of **2e**

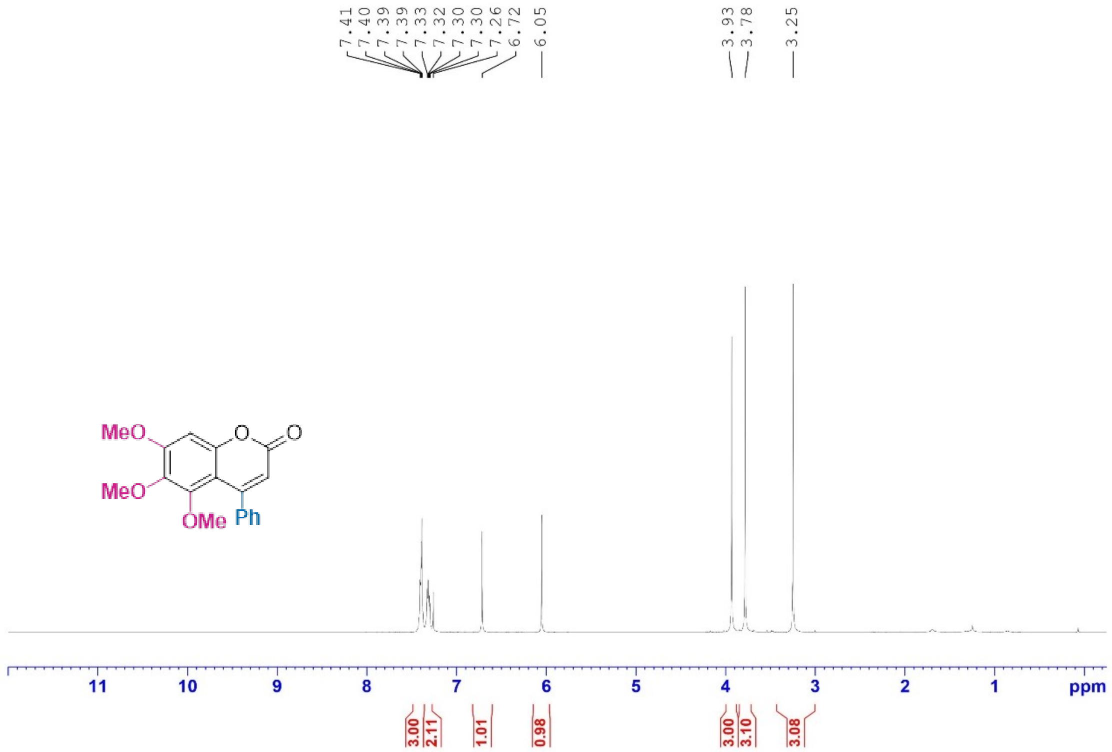

^{1}H NMR (300 MHz, CDCl₃) spectrum of **2g**

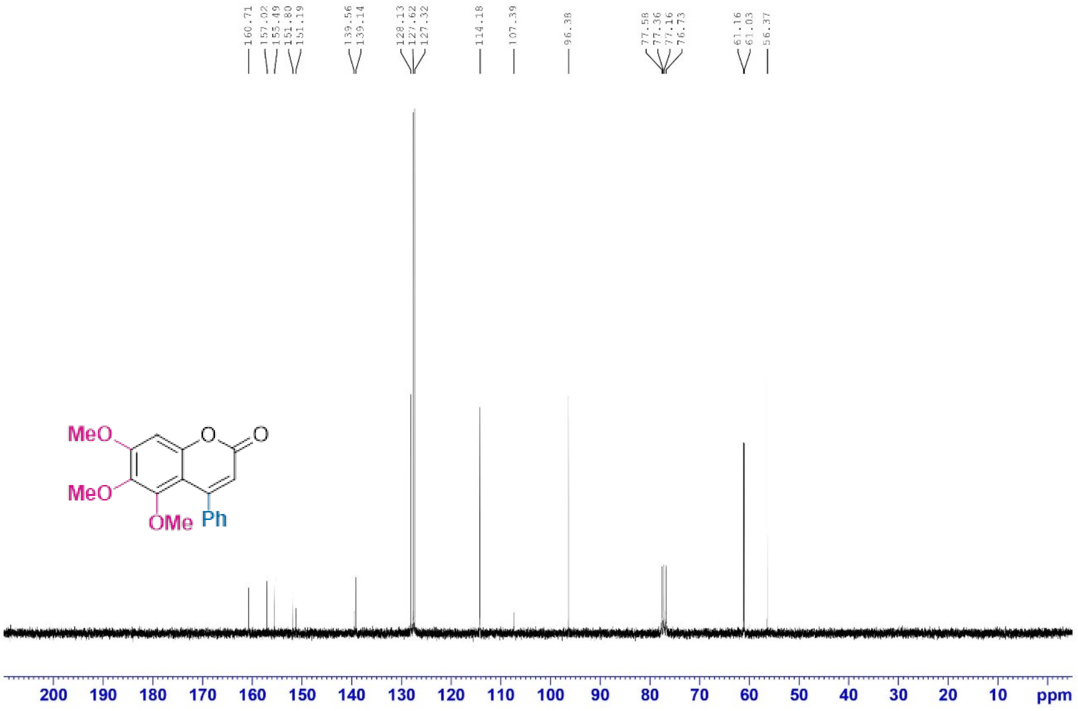

^{13}C{^{1}H} NMR (77.5 MHz, $CDCl_3$) spectrum of **2g**

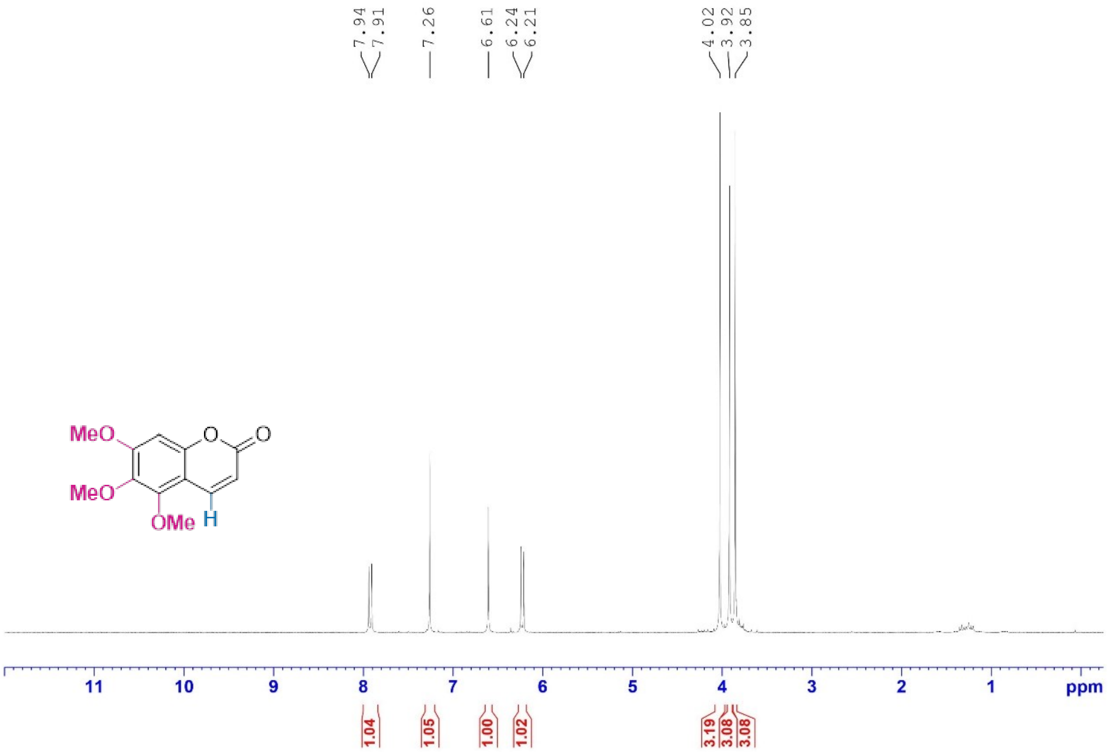

^{1}**H NMR (300 MHz, CDCl$_3$)** spectrum of **2h**

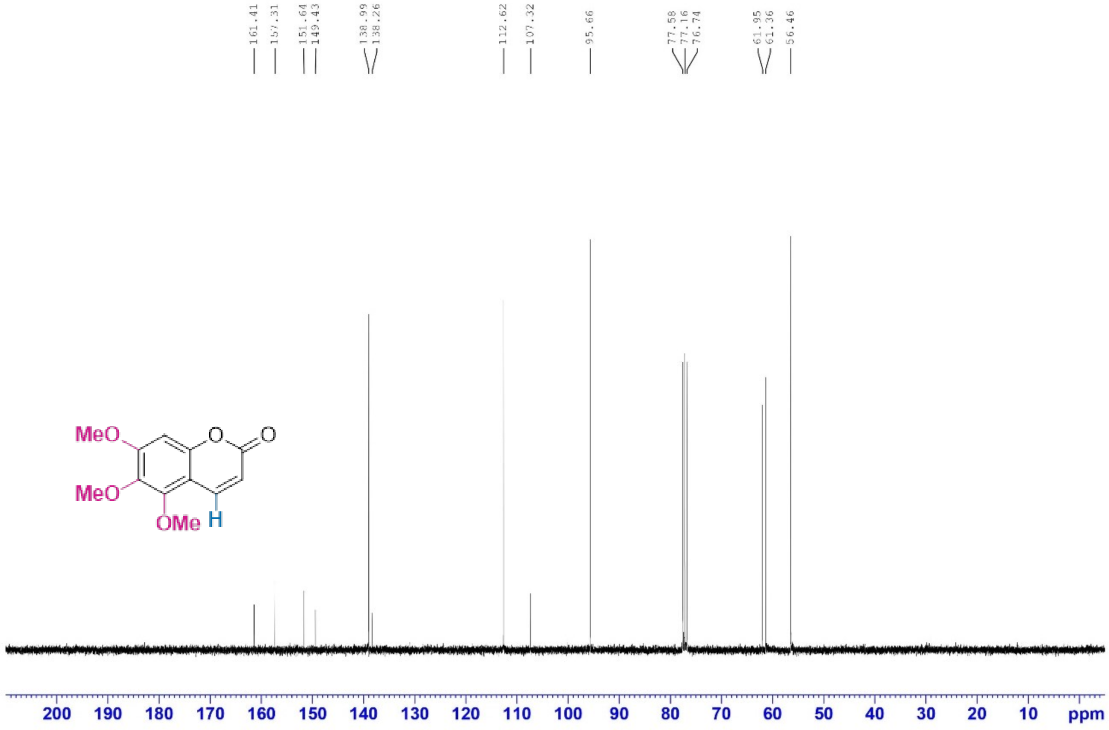

^{13}**C{^{1}H} NMR (77.5 MHz, CDCl$_3$)** spectrum of **2h**

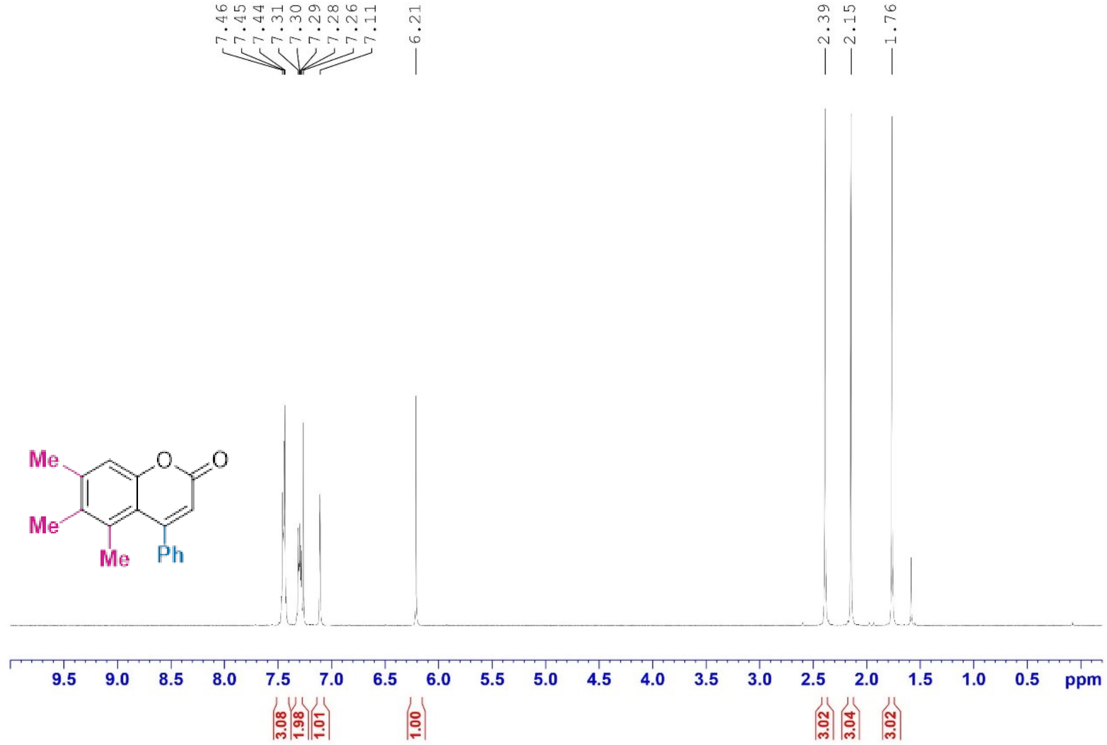

^{1}H NMR (300 MHz, $CDCl_3$) spectrum of **2i**

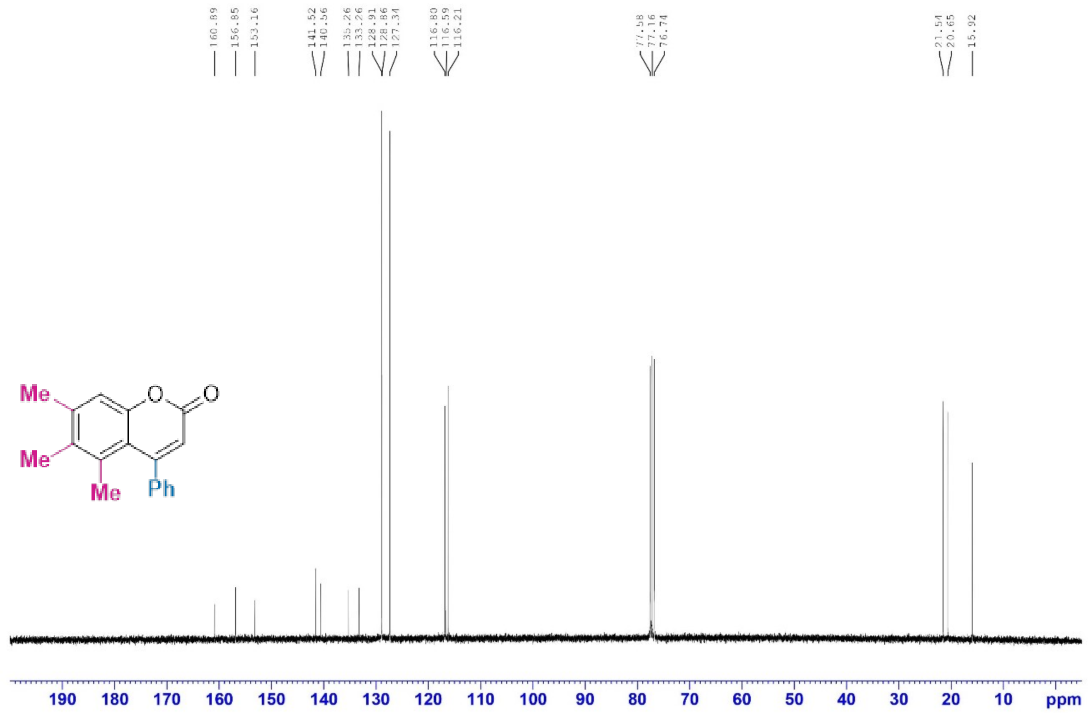

^{13}C{^{1}H} NMR (77.5 MHz, $CDCl_3$) spectrum of **2i**

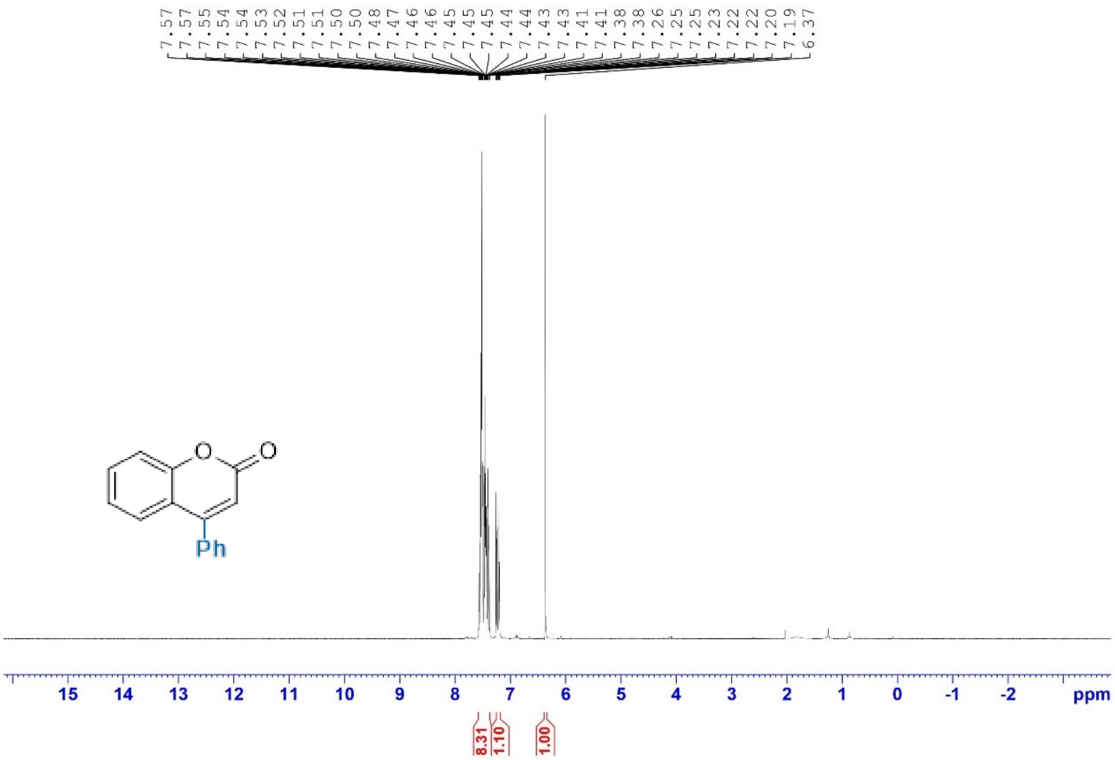

^{1}H NMR (300 MHz, CDCl₃) spectrum of **2j**

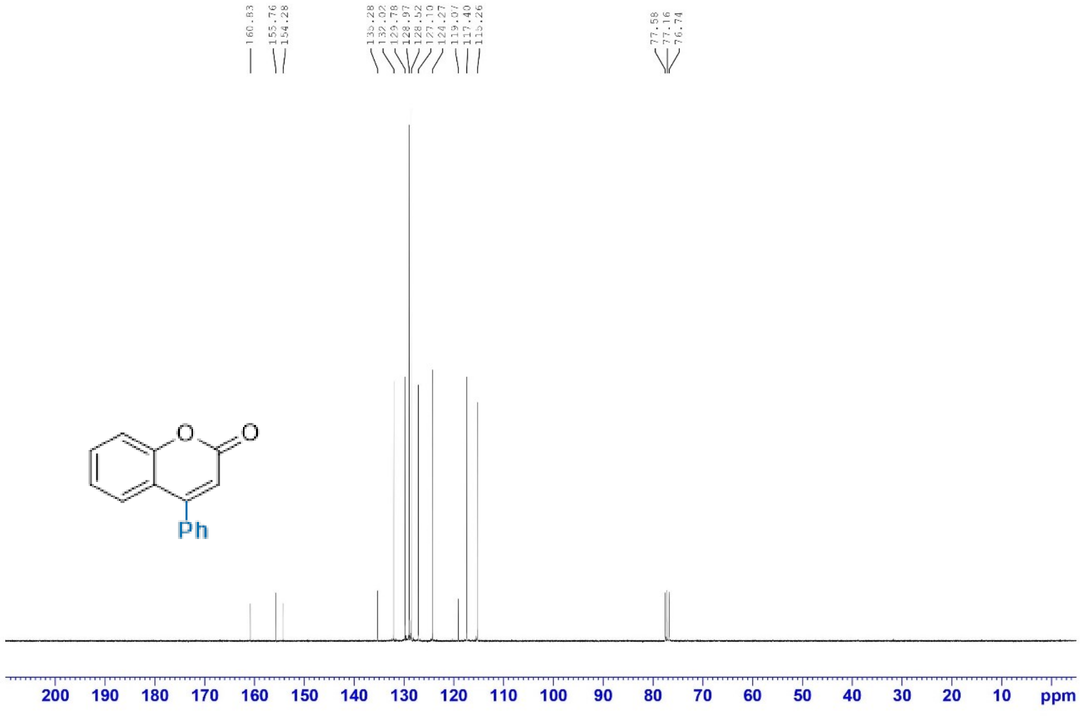

^{13}C{^{1}H} NMR (77.5 MHz, CDCl₃) spectrum of **2j**

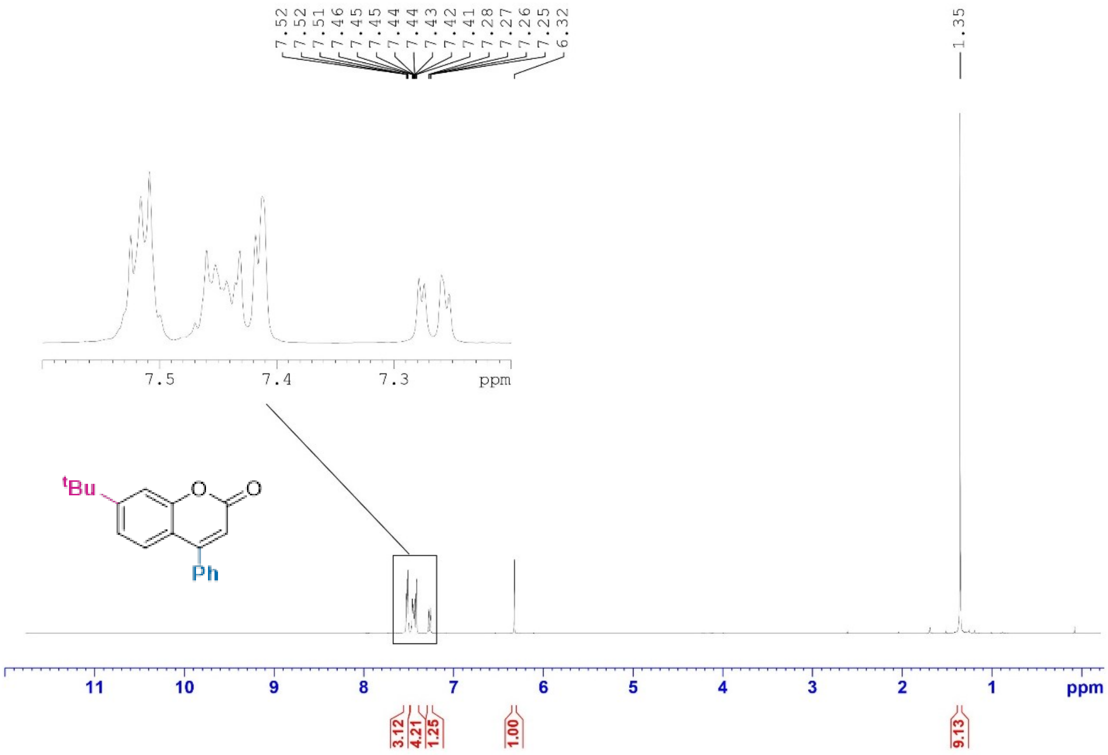

^{1}H NMR (400 MHz, $CDCl_3$) spectrum of **2k**

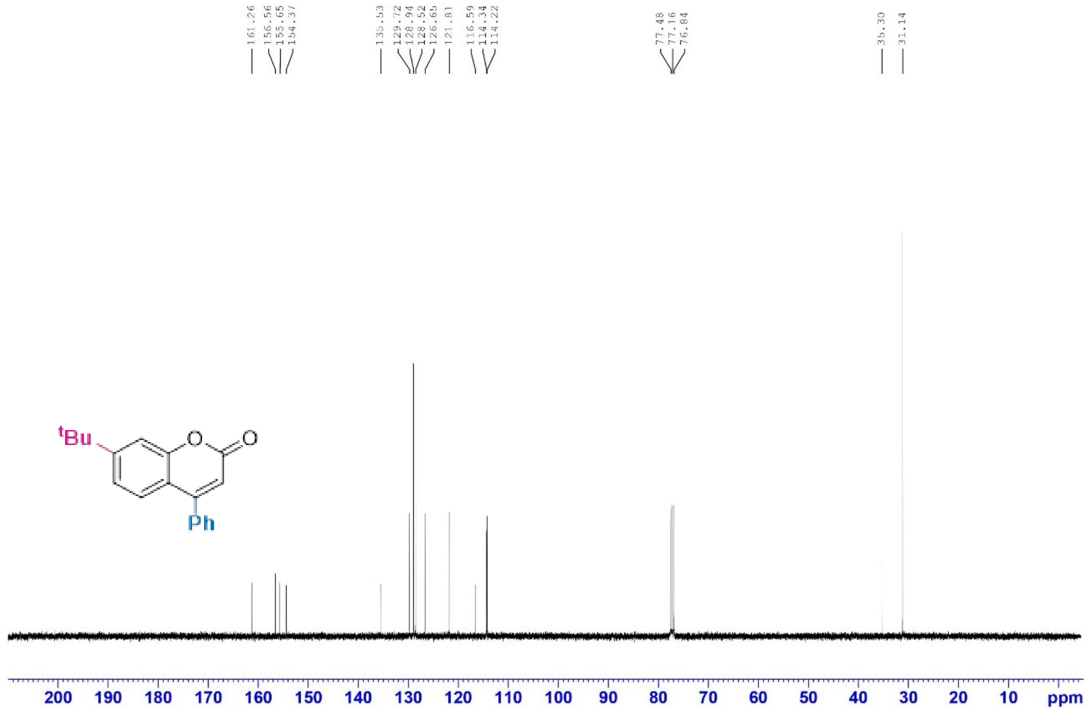

$^{13}C\{^{1}H\}$ NMR (100.6 MHz, $CDCl_3$) spectrum of **2k**

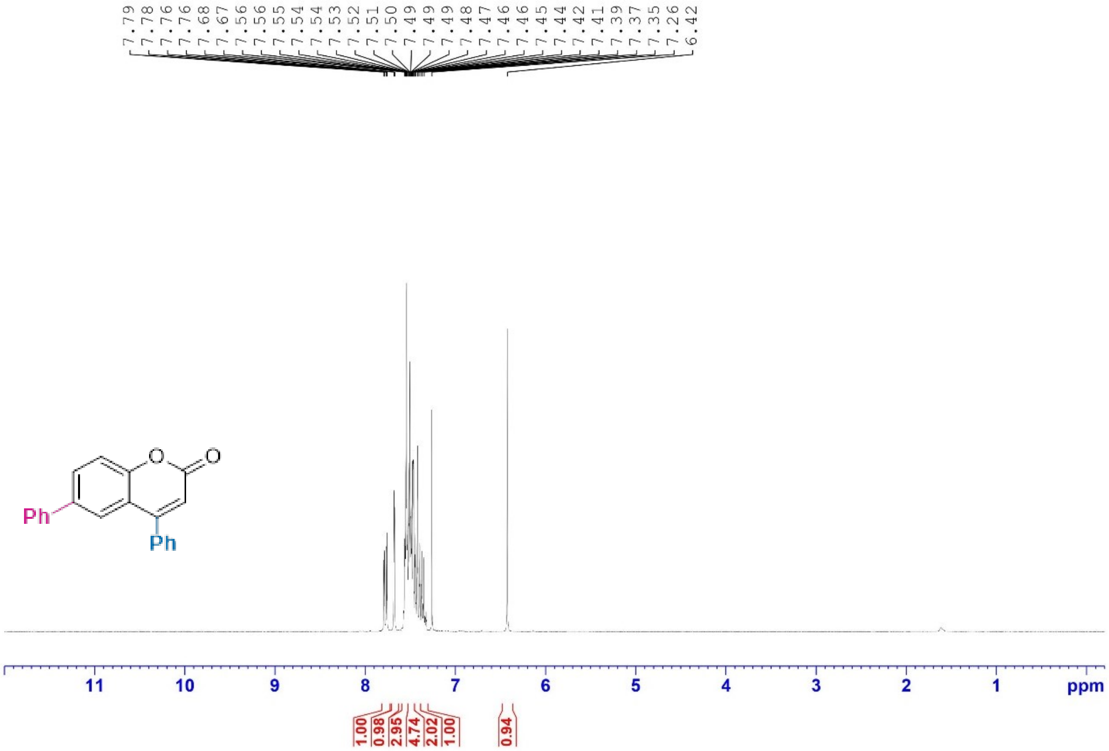

^{1}H NMR (300 MHz, CDCl$_3$) spectrum of **2m**

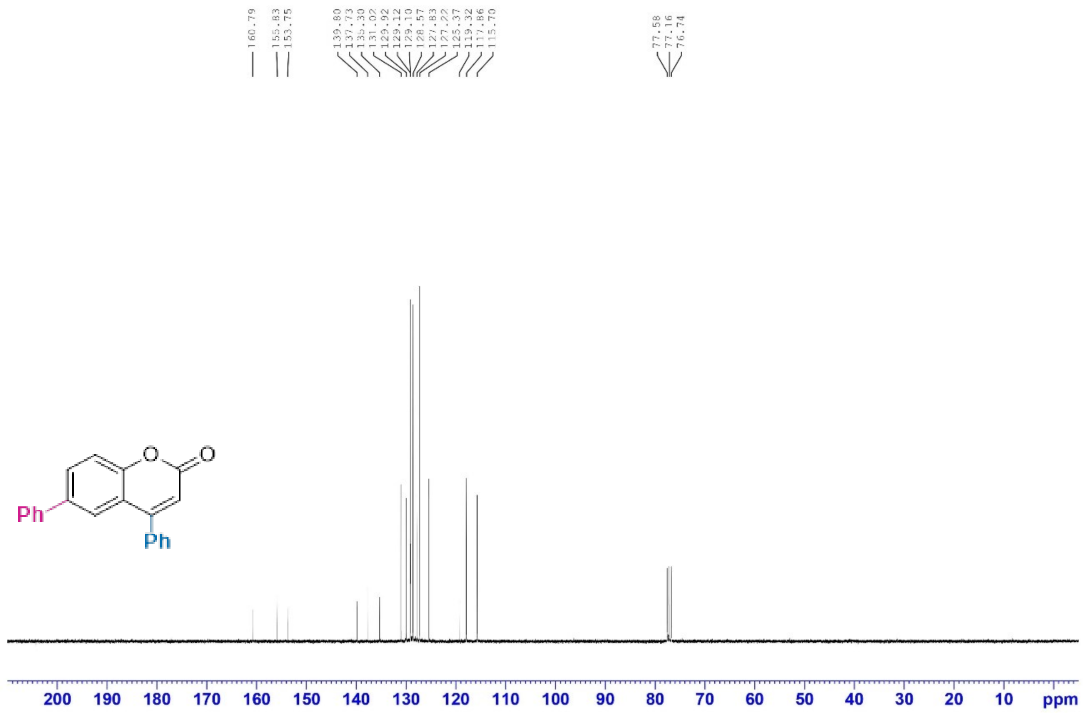

^{13}C{^{1}H} NMR (77.5 MHz, CDCl$_3$) spectrum of **2m**

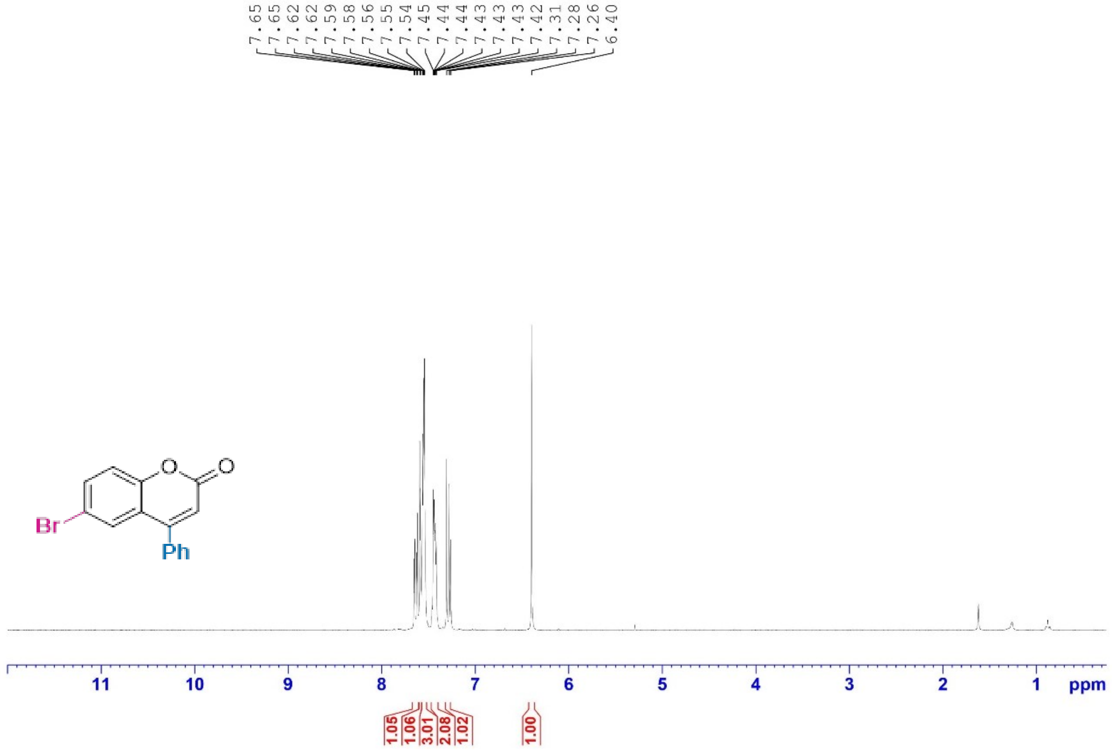

^{1}H NMR (300 MHz, CDCl$_3$) spectrum of **2n**

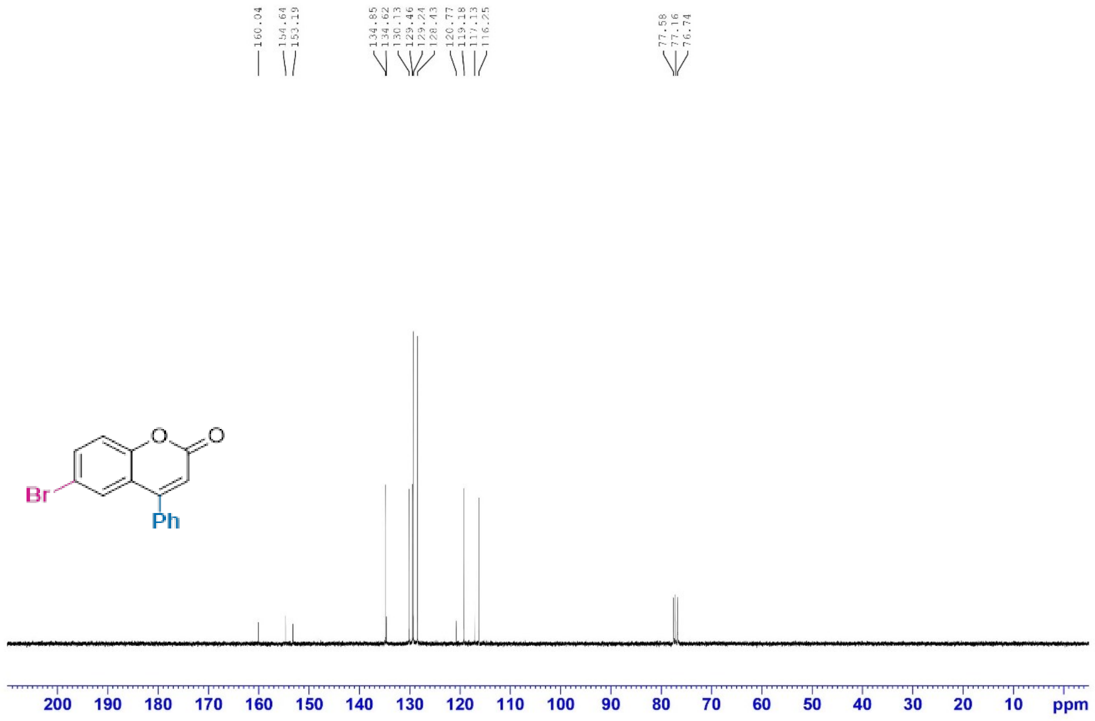

^{13}C{^{1}H} NMR (77.5 MHz, CDCl$_3$) spectrum of **2n**
