# Supplementary material for: An Improved Gold(I) Catalytic System for the Preparation of Coumarins via Intramolecular Cyclization
Source: Chem Asian J. 2024 Nov 9;20(1):e202400725. doi: 10.1002/asia.202400725 (PMC11697228; doi:10.1002/asia.202400725)
Supplement: Supplementary file 2 — Supporting Information [file ASIA-20-e202400725-s001.pdf]

Chemistry - An Asian Journal

Supporting Information

An Improved Gold(I) Catalytic System for the Preparation of Coumarins *via* Intramolecular Cyclization

Francesco Ravera, Federico Floreani, Cristina Tubaro, Marco Roverso, Riccardo Pedrazzani, Marco Bandini, and Andrea Biffis*

An Improved Gold(I) Catalytic System for the Preparation of Coumarins via Intramolecular Cyclization

Supporting Information

F. Ravera,[a,b] F. Floreani,[a,b] C. Tubaro,[a,b] M. Roverso,[a] R. Pedrazzani,[c] M. Bandini,[c] A. Biffis [a,b]

[a] Department of Chemical Sciences − DiSC, University of Padova, via Marzolo 1, 35131 Padova, Italy.

[b] Consorzio Interuniversitario per le Reattività Chimiche e la Catalisi (CIRCC), c/o Department of Chemical Sciences − DiSC, University of Padova, via Marzolo 1, 35131 Padova, Italy.

[c] Department of Chemistry "G. Ciamician", Alma Mater Studiorum − University of Bologna, Via Selmi 2, I-40126 Bologna, Italy.

Contents

Synthesis of aryl alkynoates S1
Synthesis of the catalyst ImPyAuCl S6
General procedure for catalytic test of Table 1 .... S7
General procedure for catalytic test of Table 2 .... S7
General procedures for hydroarylation reactions S8
Bibliography S13

General remarks

All the experimental manipulations were carried out under an argon atmosphere, using standard Schlenk techniques and flame-dried glassware. With the exception of the alkyne 3-(4-methyl-phenyl)-2-propynoic acid, all the other starting materials were purchased as high purity reagents and used as received. Ionic liquids were purchased from IoLiTec at high purity grade (>99%). Purifications were performed by flash-column chromatography on silica gel and TLC check for selecting the eluent and monitoring the isolated fractions.

^{1}H, ^{13}C{^{1}H} and ^{19}F NMR spectra were recorded on a Bruker Avance 200, 300 and 400 MHz spectrometers at 298 K; the ^{1}H and ^{13}C chemical shifts (δ) were calibrated with respect to signals of the deuterated solvent (^{13}C) and its protonated residue (^{1}H).[1,2] Chemical shifts in ^{19}F spectra were calibrated indirectly.[3] The following abbreviations and their combinations are used for the compressed notation of the characterization data: br, broad; s, singlet; d, doublet; t, triplet; q, quartet; m, multiplet; ses, sextet; hept, heptuplet. Data elaboration was performed with the software TopSpin 4.2.0.

HRMS data were recorded in positive mode on a Thermo Q-Exactive™ mass spectrometer equipped with an ESI source. Samples were dissolved in CH_2Cl_2 (DCM, HPLC) prior to analysis. The software for analysis of MS data was Xcalibur 4.7 (Thermo Fisher Scientific).

Synthesis of aryl alkynoates

Aryl alkynoates were prepared according to a previously reported procedure:[4]

In a round-bottom flask, a stirred solution of the phenol (3.0 mmol, 1.0 eq.) in DCM (12 ml) was cooled to 0°C and the propiolic acid* derivative (3.3 mmol, 1.1 eq.) was added. Subsequently, a mixture of dicyclohexylcarbodiimide (DCC, 873.0 mg, 4.4 mmol, 1.5 eq.) and 4-dimethylaminopyridine (DMAP, 36.0 mg, 0.3 mmol, 0.1 eq.) in DCM (6 mL) was added dropwise to the round-bottom flask. The resulting mixture was allowed to warm up and stirred at room temperature for 12 hours. Then the mixture was filtered and the solid residue on the filter was washed with DCM (15 mL). The combined organic phases were concentrated under reduced pressure and the obtained residue purified by flash chromatography over silica gel. The desired product was isolated upon evaporation of the relevant fractions.

* Propiolic acid, phenylpropiolic acid, 3-(trimethylsilyl)-2-propynoic acid, 2-butynoic acid and 2-hexynoic acid were directly purchased and used without further purification, while the derivative 3-(4'-methyl-phenyl)-2-propynic acid was synthesized according to the following procedure:[4] 4-iodotoluene (1.323 g, 6.0 mmol, 1.0 eq.), 1,8-diazabicyclo(5.4.0)undec-7-ene (DBU, 2.01 g, 13.2 mmol, 2.2 eq.), and $Pd(PPh_3)_4$ (347.1 mg, 0.3 mmol, 5 mol %) were taken in a flame dried, round bottom flask with a magnetic stirrer. The reaction tube was purged with argon and then dry DMSO (15 mL) was added via a syringe. Propiolic acid (0.40 ml, 6.6 mmol, 1.1 eq.) was added into the flask and then the mixture was stirred at room temperature for 12 h. After completion of the reaction (TLC monitored), EtOAc (10 mL) was added into the reaction mixture. The reaction mixture was extracted with saturated aqueous $NaHCO_3$. The aqueous layer was separated, acidified by addition of cold HCl (1 M) and extracted with DCM. The combined organic layers were dried with anhydrous Na_2SO_4 and filtered, and the solvent was removed under reduced pressure.

The resulting crude product was purified by column chromatography on silica gel (MeOH/DCM/AcOH 8:90:2 as eluent). The product was identified by ^{1}H NMR spectroscopy and was subsequently employed for the synthesis of the corresponding aryl alkynoate.

3’,4’-Methylendioxy-phenyl 3-phenyl-2-propynoate – 1a

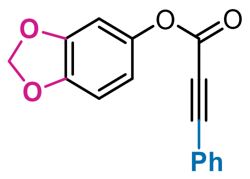

Synthesized employing sesamol and 3-phenyl-2-propynoic acid as starting materials. The corresponding alkynoate was isolated by flash chromatography (eluent EtOAc/Hex 1:10) to obtain 593 mg of product as a white solid (2.22 mmol, 74% yield). Spectroscopic data were in agreement with previously reported ones. [5]

^{1}H NMR (300 MHz, CDCl$_3$): δ 7.66–7.60 (m, 2H), 7.53–7.45 (m, 1H), 7.44–7.36 (m, 2H), 6.80 (d, 1H, *J* = 8.4 Hz), 6.71 (d, 1H, *J* = 2.4 Hz), 6.64 (dd, 1H, *J*= 8.4 Hz, *J* = 2.4 Hz,), 5.99(s, 2H) ppm.

3’,4’-Methylendioxy-phenyl 3-(4’-methylphenyl)-2-propynoate – 1b

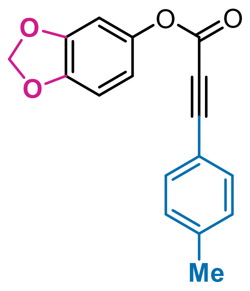

Synthesized employing sesamol and 3-(4’-methylphenyl)-2-propynoic acid as starting materials. The corresponding alkynoate was isolated by flash chromatography (eluent EtOAc/Hex 1:10) to obtain 52.6 mg of product as a pale yellow solid (0.188 mmol, 3% overall yield from 4-iodotoluene and propiolic acid*).

^{1}H NMR (300MHz, CDCl$_3$): δ 7.57–7.48 (m, *J* = 7.8 Hz, 2H), 7.26–7.16 (m, *J* = 7.8 Hz, 2H), 6.80 (d, *J* = 8.3 Hz, 1H), 6.70 (brs, 1H), 6.64 (d, *J* = 8.3 Hz, 1H), 5.99 (s, 2H), 2.40 (s, 3H) ppm; **^{13}C{^{1}H} NMR (75.5 MHz, CDCl$_3$):** δ 152.9, 148.2, 145.9, 144.5, 142.0, 133.3, 129.6, 116.2, 114.1, 108.2, 103.7, 101.9, 89.5, 80.0, 21.9 ppm; **HRMS (ESI+):** calcd for $[M+H]^+$ = $C_{17}H_{13}O_4^+$: 281.0808 m/z. Found: 281.0890 m/z.

* the low yield is mainly due to the inefficiency of the Sonoghashira coupling of 4-iodotoluene with propiolic acid.

3’,4’-Methylendioxy-phenyl-2-propynoate – 1c

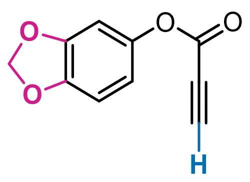

Synthesized employing sesamol and propiolic acid as starting materials. The corresponding alkynoate was isolated by flash chromatography (eluent EtOAc/Hex 1:10) to obtain 356 mg of product as a white solid (1.87 mmol, 62% yield). Spectroscopic data were in agreement with previously reported ones.[6]

^{1}H NMR (300 MHz, $CDCl_3$): δ 6.79 (d, *J* = 8.4 Hz, 1H), 6.66 (d, *J* = 2.4 Hz, 1H), 6.59 (dd, *J* = 8.4, 2.4 Hz, 1H), 5.99 (s, 2H), 3.07 (s, 1H) ppm.

3',4'-Methylendioxy-phenyl-2-butynoate – 1d

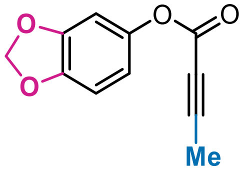

Synthesized employing sesamol (scaled-up procedure to 6 mmol) and 2-butynoic acid as starting materials. The corresponding alkynoate was isolated by flash chromatography (eluent EtOAc/Hex 1:10) to obtain 846 mg of product as a white solid (4.14 mmol, 69% yield).

^{1}H NMR (300 MHz, $CDCl_3$): δ 6.74 (d, *J* = 8.4 Hz, 1H), 6.63 (d, *J* = 2.3 Hz, 1H), 6.55 (dd, *J* = 8.4, 2.3 Hz, 1H), 5.94 (s, 2H), 2.01 (s, 3H) ppm; **^{13}C{^{1}H} NMR (75.5 MHz, $CDCl_3$):** δ 152.3, 148.2, 145.8, 144.4, 114.0, 108.1, 103.6, 101.9, 88.4, 72.1, 3.9 ppm; **HRMS (ESI+):** calcd for $[M+H]^+$ = $C_{11}H_9O_4^+$: 205.0495. Found: 205.0546.

3',4'-Methylendioxy-phenyl-2-hexynoate – 1e

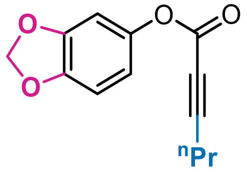

Synthesized employing sesamol and 2-hexynoic acid as starting materials. The corresponding alkynoate was isolated by flash chromatography (eluent EtOAc/Hex 1:10) to obtain 395 mg of product as a white solid (1.70 mmol, 57% yield).

^{1}H NMR (300 MHz, $CDCl_3$): δ 6.77 (d, 1H, *J* = 8.4 Hz), 6.64 (d, 1H, *J* = 2.4 Hz), 6.57 (dd, 1H, *J* = 8.4, 2.4 Hz), 5.98 (s, 2H), 2.37 (t, 2H, *J* = 7.1 Hz), 1.65 (ses, 2H, *J* = 7.2 Hz), 1.04 (t, 3H, *J* = 7.4 Hz) ppm; **^{13}C{^{1}H} NMR (75.5 MHz, $CDCl_3$):** δ 152.5, 148.2, 145.8, 144.5, 114.0, 108.1, 103.7, 101.9, 92.4, 72.9, 21.1, 20.9, 13.6 ppm; **HRMS (ESI+):** calcd for $[M+H]^+$ = $C_{13}H_{13}O_4^+$: 233.0808 m/z. Found: 233.0844 m/z.

3',4'-Methylendioxy-phenyl-3-trimethylsilyl-2-propynoate – 1f

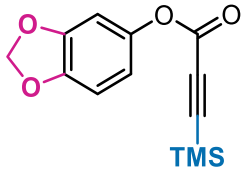

Synthesized employing sesamol (scaled-up procedure to 6 mmol) and 3-trimethyl-silyl-2-propynoic acid as starting materials. The corresponding alkynoate was isolated by flash chromatography (eluent EtOAc/Hex 1:10) to obtain 393 mg of product as a colorless oil (1.50 mmol, 25 % yield).

^{1}H NMR (300MHz, $CDCl_3$): δ 6.76 (d, *J* = 8.4 Hz, 1H), 6.64 (d, *J* = 2.4 Hz, 1H), 6.57 (dd, *J* = 8.4, 2.4 Hz, 1H), 5.97 (s, 2H), 0.28 (s, 9H) ppm; **^{13}C{^{1}H} NMR (75.5 MHz, $CDCl_3$):** δ 151.6, 148.2, 145.9, 144.2, 113.9, 108.1, 103.6, 101.9, 96.9, 94.0, -0.8 ppm; **HRMS**

(ESI+): calcd for $[M+H]^+$ = $C_{13}H_{15}O_4Si^+$: 263.0734 m/z. Found: 263.0772 m/z.

3',4',5'-Trimethoxy-phenyl-3-phenyl-2-propynoate – 1g

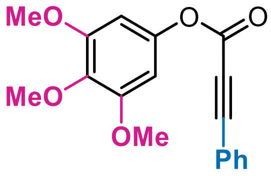

Synthesized employing 3,4,5-trimethoxyphenol and 3-phenyl-2-propynoic acid as starting materials. The corresponding alkynoate was isolated by flash chromatography (eluent EtOAc/Hex 1:5) to obtain 561 mg of product as a yellow solid (1.80 mmol, 60% yield). Spectroscopic data were in agreement with previously reported ones.[7]

^{1}H NMR (300 MHz, $CDCl_3$): δ 7.67–7.58 (m, 2H), 7.54–7.46 (m, 1H), 7.45-7.36 (m, 2H), 6.44 (s, 2H), 3.85 (s, 6H), 3.84 (s, 3H) ppm.

3',4',5'-Tri-methoxy-phenyl-3-phenyl-2-propynoate – 1h

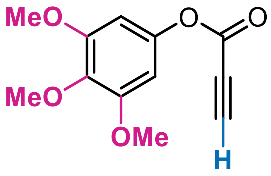

Synthesized employing 3,4,5-trimethoxy-phenol and propiolic acid as starting materials. The corresponding alkynoate was isolated by flash chromatography (eluent EtOAc/Hex 1:5) to obtain 309 mg of product as an orangish solid (1.31 mmol, 44% yield).

^{1}H NMR (300 MHz, $CDCl_3$): δ 6.38 (s, 2H), 3.83 (s, 6H), 3.82 (s, 3H), 3.09 (s, 1H) ppm; **$^{13}C\{^1H\}$ NMR (75.5 MHz, $CDCl_3$):** δ 153.7, 151.1, 145.9, 136.4, 98.9, 77.1, 74.3, 61.0, 56.3 ppm; **HRMS (ESI+):** calcd for $[M+H]^+$ = $C_{12}H_{13}O_5^+$: 237.0757 m/z. Found: 237.0785 m/z.

3',4',5'-Trimetyl-phenyl-3-phenyl-2-propynoate – 1i

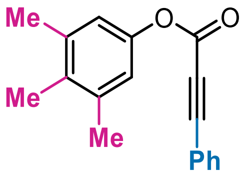

Synthesized employing 3,4,5-tri-methyl-phenol and 3-phenyl-2-propynoic acid as starting materials. The corresponding alkynoate was isolated by flash chromatography (eluent EtOAc/Hex 1:10) to obtain 384 mg of product as a white solid (1.45 mmol, 48% yield).

^{1}H NMR (300 MHz, $CDCl_3$): δ 7.69–7.61 (m, J = 7.8 Hz, 2H), 7.54–7.46 (m, 1H), 7.45–7.37 (m, 2H), 6.85 (s, 2H), 2.31 (s, 6H), 2.16 (s, 3H) ppm; **$^{13}C\{^1H\}$ NMR (75.5 MHz, $CDCl_3$):** δ 153.0, 147.3, 138.0, 133.5, 133.2, 131.0, 128.7, 120.2, 119.4, 88.3, 80.5, 20.8, 15.1 ppm; **HRMS (ESI+):** calcd for $[M+H]^+$ = $C_{18}H_{17}O_2^+$: 265.1223 m/z. Found: 265.1256 m/z.

Phenyl-3-phenyl-2-propynoate – 1j

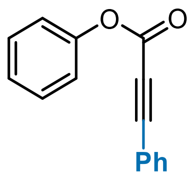

Synthesized employing phenol and 3-phenyl-2-propynoic acid as starting materials. The corresponding alkynoate was isolated by flash chromatography (eluent EtOAc/Hex 1:20) to obtain 415 mg of product as a white solid (1.87 mmol, 62% yield). Spectroscopic data were in agreement with previously reported ones.[5]

^{1}H NMR (300 MHz, $CDCl_3$): δ 7.66–7.61 (m, 2H), 7.53–7.37 (m, 5H), 7.32–7.27 (m, 1H), 7.23–7.17 (m, 2H) ppm.

3'-Tert-butyl-phenyl-3-phenyl-2-propynoate – 1k

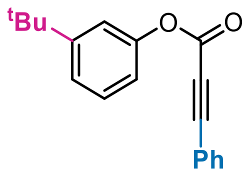

Synthesized employing 3-tert-butyl-phenol and 3-phenyl-2-propynoic acid as starting materials. The corresponding alkynoate was isolated by flash chromatography (eluent EtOAc/Hex 1:20) to obtain 366 mg of product as a greenish oil (1.31 mmol, 44% yield). Spectroscopic data were in agreement with previously reported ones.

^{1}H NMR (300 MHz, $CDCl_3$): δ 7.66–7.60 (m, 2H), 7.53–7.45 (m, 1H), 7.44–7.37 (m, 2H), 7.34 (d, *J* = 7.6 Hz, 1H), 7.31 (dt, *J* = 7.8, 1.6 Hz, 1H), 7.19 (t, *J* = 1.9 Hz, 1H), 7.03 (dt, *J* = 7.5, 1.9 Hz, 1H), 1.34 (s, 9H) ppm; **$^{13}C\{^1H\}$ NMR (75.5 MHz, $CDCl_3$):** δ 153.5, 152.6, 150.2, 133.3, 131.1, 129.2, 128.8, 123.6, 119.5, 118.6, 88.7, 80.5, 35.0, 31.4 ppm; **HRMS (ESI+):** calcd for $[M+H]^+$ = $C_{19}H_{19}O_2^+$: 279.1380 m/z. Found: 279.1408 m/z.

2'-Methoxy-phenyl 3-phenyl-2-propynoate – 1l

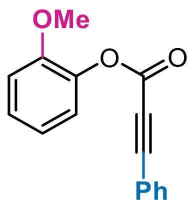

Synthesized employing guaiacol and 3-phenyl-2-propynoic acid as starting materials. The corresponding alkynoate was isolated by flash chromatography (eluent EtOAc/Hex 1:5) to obtain 401 mg of product as a pale yellow solid (1.59 mmol, 53% yield). Spectroscopic data were in agreement with previously reported ones. [8]

^{1}H NMR (300MHz, $CDCl_3$): δ 7.66–7.58 (m, 2H), 7.52–7.44 (m, 1H), 7.43–7.35 (m, 2H), 7.30–7-22 (m, 1H), 7.20–7.13 (m, 1H), 7.05–6.95 (m, 2H), 3.85 (s, 3H) ppm.

Biphenyl-4'-yl-3-phenyl-2-propynoate – 1m

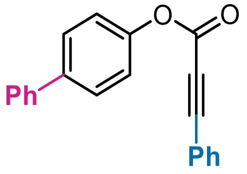

Synthesized employing 4-phenyl-phenol and 3-phenyl-2-propynic acid as starting materials. The corresponding alkynoate was isolated by flash chromatography (eluent Toluene/Hex 1:1) to obtain 608 mg of product as a white solid (2.04 mmol, 68% yield). Spectroscopic data were in agreement with previously reported ones.[9]

^{1}H NMR (300 MHz, $CDCl_3$): δ 7.68–7.60 (m, 5H), 7.60–7.55 (m, 2H), 7.55–7.33 (m, 6H), 7.31–7.24 (m, 2H) ppm.

4'-Bromo-phenyl-3-phenyl-2-propynoate – 1n

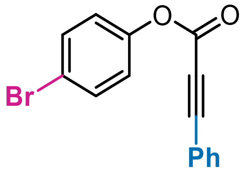

Synthesized employing 4-bromo-phenol and 3-phenyl-2-propynoic acid as starting materials. The corresponding alkynoate was isolated by flash chromatography (eluent EtOAc/Hex 1:10) to obtain 738 mg of product as a white solid (2.45 mmol, 82% yield). Spectroscopic data were in agreement with previously reported ones.[9]

^{1}H NMR (300 MHz, $CDCl_3$): δ 7.67–7.60 (m, 2H), 7.57–7.51 (m, *J* = 8.8 Hz, 2H), 7.51–7.46 (m, 1H), 7.46–7.37 (m, 2H), 7.14–7.06 (m, *J* = 8.8 Hz, 2H) ppm.

4'-Nitro-phenyl-3-phenyl-2-propynoate – 1o

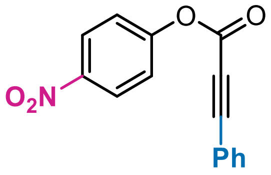

Synthesized employing 4-bromo-phenol and 3-phenyl-2-propynoic acid as starting materials. The corresponding alkynoate was isolated by flash chromatography (eluent EtOAc/Hex 1:10) to obtain 571 mg of product as a pale yellow solid (2.14 mmol, 71% yield). Spectroscopic data were in agreement with previously reported ones.[8]

^{1}H NMR (400 MHz, $CDCl_3$): δ 8.34-8.29 (m, *J* = 9.2 Hz, 2H), 7.68-7.64 (m, 2H), 7.56-7.50 (m, 1H), 7.47-7.38 (m, 4H) ppm.

Synthesis of the catalyst ImPyAuCl

The synthesis has been accomplished according to a reported literature procedure.[10] In a flame-dried two-necked round bottom flask filled with argon the imidazolium salt ImPy·HCl (52.2 mg, 0.10 mmol, 1 eq.) was dissolved in 1 mL of anhydrous DCM and then Ag_2O (25.6 mg, 0.11 mmol, 1.1 eq.) was added: aluminum foils were used to protect the reaction mixture from light. The reaction mixture was stirred at room temperature overnight. TLC monitoring using

EtOAc/^{c}Hex 1:2 was performed to identify formation of the respective NHC-Ag complex. The reaction was filtered through a Celite® pad followed by DCM to rinse the filter. The organic phase was then evaporated, taken up in 1 mL of anhydrous DCM and transferred inside a two-necked round-bottom flask under inert atmosphere (aluminum foils were employed also in this step). [AuCl(SMe$_2$)] (29.6 mg, 0.10 mmol, 1 eq.) was then added and the solution was stirred at room temperature for 5 h. After the silver precursor was completely consumed, the reaction crude was filtered through a Celite® pad, the filter was rinsed with DCM and all volatiles were removed in *vacuo*. The resulting yellowish solid was recrystallized by layering n-hexane over a concentrated solution of DCM, to afford 49.1 mg of the desired ImPyAuCl complex (0.068 mmol, 68% yield). The collected spectroscopic data were in accordance with previously reported ones.[10]

ImPyAuCl

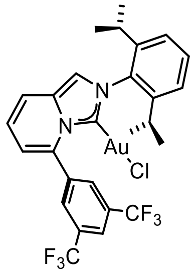

^{1}H NMR (300 MHz, CDCl$_3$): δ = 8.11 – 7.97 (m, 3H), 7.57 (dd, *J* = 9.3, 0.9 Hz, 1H), 7.47 (t, *J* = 7.8 Hz, 1H), 7.41 (s, 1H), 7.26 – 7.21 (m, 2H), 7.12 (dd, *J* = 9.3, 6.7 Hz, 1H), 6.73 (dd, *J* = 6.7, 0.9 Hz, 1H), 2.16 (hept, *J* = 6.6 Hz, 2H), 1.25 (d, *J* = 6.8 Hz, 6H), 1.13 (d, *J* = 6.6 Hz, 6H) ppm; **^{19}F NMR (188 MHz, CDCl$_3$):** δ -62.92 ppm.

General procedure for catalytic test of Table 1

The substrate **1a** (133.1, 0.5 mmol) was placed in a flame-dried Schlenk tube under argon atmosphere. The gold catalyst IPrAuNTf$_2$ (2.2 mg, 0.0025 mmol) was added to the flask along with 0.75 ml of a suitable ionic liquid. The internal standard, dimethylsulfone or 1,2-dimethoxyethane, (0.25 mmol) was then added to the same flask and the mixture was stirred at room temperature for five minutes. At this point a ^{1}H NMR check was taken, and the Schlenk tube was then moved in a thermostatic bath at 40 or 50 °C. The reaction kinetic was followed by sampling aliquots of the reaction crude at different times and analyzing them by ^{1}H NMR.

General procedure for catalytic test of Table 2

Different amounts of IPrAuNTf$_2$ catalyst (0.1, 0.05 or 0.01 mol% with respect to **1a**) were deposit through a titled solution of the complex in DCM.* The same procedure as for **Table 1** was used: in particular, [BMIM][NTf$_2$] was employed as ionic liquid. The acidic additive HBF$_4$·Et$_2$O (14 µl, 0.1 mmol) was added to the reaction mixture as the first ^{1}H NMR check was collected. The Schlenk tube was then placed in a thermostatic bath at 40°C and the reaction kinetic was monitored as above.

*DCM was previously treated with basic alumina in order to remove acid traces.

General procedures for hydroarylation reactions

- Conditions **A**:

The substrate (0.5 mmol) was placed in a flame-dried Schlenk tube under argon atmosphere. A freshly prepared solution of the gold catalyst $IPrAuNTf_2$ (4.3 mg, 0.005 mmol) in $[BMIM][NTf_2]$ (50 µl) was added to the flask (50 µl, 0.25 µmol, 0.05 mol% [Au] loading), along with 0.7 ml of $[BMIM][NTf_2]$ and the mixture was stirred for 5 min at room temperature. The acid co-catalyst $HBF_4·Et_2O$ (7 µl, 0.05 mmol) was then added to the mixture and the flask was placed in a thermostatic bath at 40 °C for 24 h. A ^1H NMR check was then taken to confirm the presence of products. The reaction crude was then subjected to column chromatography purification without any pre-treatment to afford the spectroscopically pure coumarin upon collection of the relevant fractions.

- Conditions **B**:

The substrate (0.5 mmol) and the gold catalyst $IPrAuNTf_2$ or ImPyAuCl (0.0025 mmol) were placed in a flame-dried Schlenk tube under argon atmosphere. The ionic liquid $[BMIM][NTf_2]$ (0.75 ml) was added to the flask and the mixture was stirred for 5 min at room temperature.* The acid co-catalyst $HBF_4·Et_2O$ (7 µl, 0.05 mmol) was then added to the mixture and the flask was placed in a thermostatic bath at 80 °C for 24 h. A ^1H NMR check was then taken to confirm the presence of products. The reaction crude was then subjected to column chromatography purification without any pre-treatment to afford the spectroscopically pure coumarin upon collection of the relevant fractions.

* When ImPyAuCl was employed, 0.1 ml of $AgSbF_6$ solution in $[BMIM][NTf_2]$ (8.6 mg $AgSbF_6$, 0.025 mmol, in 1 ml of ionic liquid) was added to remove the chloride ligand. The mixture volume was then diluted to 0.75 ml with fresh $[BMIM][NTf_2]$.

6,7-Methylendioxy-4-phenyl-2H-chromen-2-one – 2a

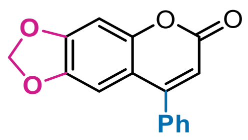

Compound **2a** was synthesized from **1a** according to general conditions **A** and purified by column chromatography (eluent EtOAc/Hex 1:4) to afford 131.3 mg of the corresponding coumarin as a white solid (0.49 mmol , 99% yield). Spectroscopic data were in agreement with previously reported ones.[11]

^{1}H NMR (300 MHz, $CDCl_3$): δ 7.52–7.50 (m, 3H), 7.45–7.39 (m, 2H), 6.88 (s, 1H), 6.82 (s, 1H), 6.23 (s, 1H), 6.05 (s, 2H) ppm; **^{13}C{^{1}H} NMR (75.5 MHz, $CDCl_3$):** δ 161.3, 156.0, 151.5, 151.3, 144.9, 135.8, 129.7, 129.0, 128.3, 113.0, 112.3, 104.5, 102.5, 98.7 ppm.

6,7-Methylendioxy-4-(4'-methylphenyl)-2H-chromen-2-one – 2b

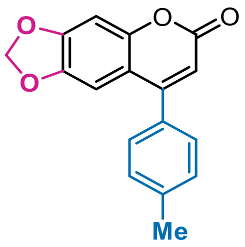

Compound **2b** was synthesized in lower scale from **1b** (0.1mmol), according to general conditions **A** and purified by column chromatography to afford 27.1 mg of the corresponding coumarin as a white solid (0.097 mmol, 97% yield).

^{1}H NMR (300MHz, $CDCl_3$): δ 7.35-7.27 (m, 4H), 6.88 (s, 1H), 6.86 (s, 1H), 6.21 (s, 2H), 6.04 (s, 2H), 2.44 (s, 3H) ppm; **^{13}C{^{1}H} NMR (75.5 MHz, $CDCl_3$):** δ 161.4, 156.1, 151.5, 151.2, 144.9, 139.9, 132.9, 129.7, 128.3, 113.1, 112.1, 104.6, 102.5, 98.7, 21.5 ppm; **HRMS (ESI+):** calcd for $[M+H]^+$ = $C_{17}H_{13}O_4^+$: 281.0808 m/z. Found: 281.0878 m/z.

6,7-Methylendioxy-2H-chromen-2-one – 2c

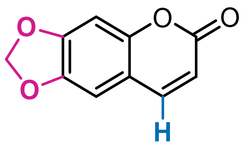

Compound **2c** was synthesized from **1c** according to general conditions **A** and purified by column chromatography (eluent DCM) to afford 80.6 mg of the corresponding coumarin as a white solid (0.42 mmol, 85% yield). Spectroscopic data were in agreement with previously reported ones.[6]

^{1}H NMR (300 MHz, $CDCl_3$): δ 7.58 (d, *J* = 9.5 Hz, 1H), 6.83 (s, 2H), 6.28 (d, *J* = 9.5 Hz, 1H), 6.07 (s, 2H) ppm; **^{13}C{^{1}H} NMR (75.5 MHz, $CDCl_3$):** δ 161.3, 151.4, 151.4, 145.0, 143.6, 113.5, 112.8, 105.1, 102.4, 98.5 ppm.

6,7-Methylendioxy-4-methyl-2H-chromen-2-one – 2d

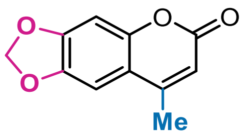

Compound **2d** was synthesized from **1d** according to general conditions **A** and purified by column chromatography (eluent DCM) to afford 95.3 mg of the corresponding coumarin as a white solid (0.47 mmol, 93% yield).

^{1}H NMR (400 MHz, $CDCl_3$): δ 6.92 (s, 1H), 6.77 (s, 1H), 6.12 (d, *J* = 0.8 Hz, 1H), 6.05 (s, 2H), 2.34 (d, *J* = 0.8 Hz, 3H) ppm; **^{13}C{^{1}H} NMR (100.6 MHz, $CDCl_3$):** δ 161.3, 152.5, 151.0, 150.6, 145.0, 113.9, 112.3, 102.4, 102.2, 98.4, 19.2 ppm; **HRMS (ESI+):** calcd for $[M+H]^+$ = $C_{11}H_9O_4^+$: 205.0495 m/z. Found: 205.0538 m/z.

6,7-Methylendioxy-4-n-propyl-2H-chromen-2-one – 2e

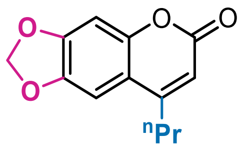

Compound **2e** was synthesized from **1e** according to general conditions **A** and purified by column chromatography (eluent EtOAc/Hex 1:4) to afford 115.2 mg of the corresponding coumarin as a brownish solid (0.5 mmol, 99% yield). Spectroscopic data were in agreement with previously reported ones.[12]

^{1}H NMR (300 MHz, $CDCl_3$): δ 6.97 (s, 1H), 6.81 (s, 1H), 6.13 (t, *J* = 0.8 Hz, 1H), 6.05 (s, 2H), 2,64 (td, *J* = 7.6, 0.8 Hz, 2H), 1.70 (ses, *J* = 7.6 Hz, 2H), 1.03 (t, *J* = 7.4 Hz, 3H) ppm; **^{13}C{^{1}H} NMR (75.5 MHz, $CDCl_3$):** δ 161.7, 156.4, 151.0, 151.0, 145.1, 113.3, 111.3, 102.4, 102.0, 98.7, 34.4, 21.5, 14.0 ppm.

5,6,7-Trimethoxy-4-phenyl-2H-chromen-2-one – 2g

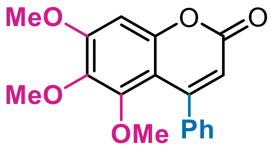

Compound **2g** was synthesized from **1g** according to general conditions **A** and purified by column chromatography (gradient EtOAc/Hex 1:5 to 1:2) to afford 139.2 mg of the corresponding coumarin as a yellow solid (0.45 mmol, 89% yield). Spectroscopic data were in agreement with previously reported ones.[7]

^{1}H NMR (300 MHz, $CDCl_3$): δ 7.49-7.36 (m, 3H),7.36-7.27 (m, 2H), 6.72 (s, 1H), 6.05 (s, 1H), 3.93 (s, 3H), 3.78 (s, 3H), 3.25 (s, 3H) ppm; **^{13}C{^{1}H} NMR (75.5 MHz, $CDCl_3$):** δ 160.7, 157.0, 155.5, 151.8, 151.2, 139.6, 139.1, 128.1, 127.6, 127.3, 114.2, 107.4, 96.4, 61.2, 61.0, 56.4 ppm.

5,6,7-Trimethoxy-2H-chromen-2-one – 2h

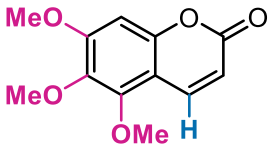

Compound **2h** was synthesized from **1h** according to general conditions **A** and purified by column chromatography (gradient EtOAc/Hex 1:5 to 1:2) to afford 108.6 mg of the corresponding coumarin as a yellow solid (0.46 mmol, 92% yield). Spectroscopic data were in agreement with previously reported ones.[13]

^{1}H NMR (300 MHz, $CDCl_3$): δ 7.92 (d, *J* = 9.6 Hz, 1H), 6.61 (s, 1H), 6.22 (d, *J* = 9.6Hz, 1H), 4.02 (s, 3H), 3.92 (s, 3H), 3.85 (s, 3H) ppm; **^{13}C{^{1}H} NMR (75.5 MHz, $CDCl_3$):** δ 161.4, 157.3, 151.6, 149.4, 139.0, 138.3, 112.6, 107.3, 95.7, 61.9, 61.4, 56.5 ppm.

5,6,7-Trimethyl-4-phenyl-2H-chromen-2-one – 2i

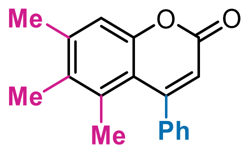

Compound **2i** was synthesized from **1i** according to general conditions **A** and purified by column chromatography (eluent EtOAc/Hex 1:4) to afford 113.3 mg of the corresponding coumarin as a dark yellow solid (0.43 mmol, 86% yield).

^{1}H NMR (300 MHz, CDCl$_3$): δ 7.48–7.41 (m, 3H), 7.34–7.27 (m, 2H), 7.11 (s, 1H), 6.21 (s, 1H), 2.39 (s, 3H), 2.15 (s, 3H), 1.76 (s, 3H) ppm; **^{13}C{^{1}H} NMR (75.5 MHz, CDCl$_3$):** δ 160.9, 156.9, 153.2, 141.5, 140.6, 135.3, 133.3, 128.9, 128.9, 127.3, 116.8, 116.6, 116.2, 21.5, 20.7, 15.9 ppm; **HRMS (ESI+):** calcd for $[M+H]^+$ = $C_{18}H_{17}O_2^+$: 265.1223 m/z. Found: 265.1249 m/z.

4-Phenyl-2H-chromen-2-one – 2j

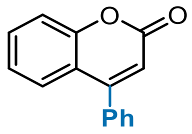

Compound **2j** was synthesized from **1j** according to general conditions **B** (IPrAuNTf$_2$ was employed as the catalyst) and purified by column chromatography (eluent EtOAc/Hex 1:8) to afford 100.3 mg of the corresponding coumarin as a white solid (0.45 mmol, 90% yield). Spectroscopic data were in agreement with previously reported ones.[14]

^{1}H NMR (300 MHz, CDCl$_3$): δ 7.58–7.40 (m, 8H), 7.26–7.21 (m, 1H), 6.37 (s, 1H) ppm; **^{13}C{^{1}H} NMR (75.5 MHz, CDCl$_3$):** δ 160.8, 155.8, 154.3, 135.3, 132.0, 129.8, 129.0, 128.5, 127.1, 124.3, 119.1, 117.4, 115.3 ppm.

7-Tert-butyl-4-phenyl-2H-chromen-2-one – 2k

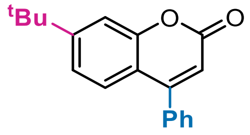

Compound **2k** was synthesized from **1k** according to general conditions **B** (IPrAuNTf$_2$ was employed as the catalyst) and purified by column chromatography (eluent EtOAc/Hex 1:8) to afford 113.9 mg of the corresponding coumarin as a viscous whitish oil (0.41 mmol, 82% yield). Spectroscopic data were in agreement with previously reported ones.[11]

^{1}H NMR (400 MHz, CDCl$_3$): δ 7.54–7.49 (m, 3H), 7.44–7.40 (m, 4H), 7.28–7.25 (m, 1H), 6.32 (s, 1H), 1.35 (s, 9H) ppm; **^{13}C{^{1}H} NMR (100.6 MHz, CDCl$_3$):** δ 161.3, 156.6, 155.6, 154.4, 135.5, 129.7, 128.9, 128.5, 126.6, 121.8, 116.6, 114.3, 114.2, 35.3, 31.1 ppm.

4,6-Di-phenyl-2H-chromen-2-one – 2m

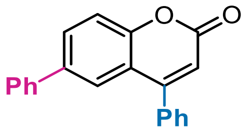

Compound **2m** was synthesized from **1m** according to general conditions **B** (ImPyAuCl was employed as the catalyst) and purified by column chromatography (eluent EtOAc/Hex 1:5) to afford 115.4 mg of the corresponding coumarin as a white solid (0.39 mmol, 77% yield). Spectroscopic data were in agreement with previously reported ones.[15]

^{1}H NMR (300 MHz, $CDCl_3$): δ 7.75 (dd, J = 8.6, 2.2 Hz, 1H), 7.66 (d, J = 2.2 Hz, 1H), 7.58–7.58 (m, 3H), 7.58–7.45 (m, 5H), 7.45–7.39 (m, 2H), 7.39–7.31 (m, 1H), 6.40 (s, 1H) ppm; **^{13}C{^{1}H} NMR (75.5 MHz, $CDCl_3$):** δ 160.8, 155.8, 153.8, 139.8, 137.7, 135.3, 131.0, 129.9, 129.1, 129.1, 128.6, 127.8, 127.2, 125.4, 119.3, 117.9, 115.7 ppm.

6-Bromo-4-phenyl-2H-chromen-2-one – 2n

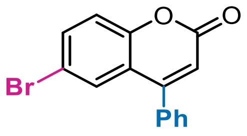

Compound **2n** was synthesized from **1n** according to general conditions **B** (ImPyAuCl was employed as the catalyst) and purified by column chromatography (eluent EtOAc/Hex 1:10) to afford 77.9 mg of the corresponding coumarin as a white solid (0.26 mmol, 52% yield). Spectroscopic data were in agreement with previously reported ones.[14]

^{1}H NMR (300 MHz, $CDCl_3$): δ 7.59–7.66 (m, 2H), 7.55–7.57 (m, 3H), 7.43–7.45 (m, 2H), 7.30 (d, J = 8.8 Hz, 1H), 6.41 (s, 1H) ppm; **^{13}C{^{1}H} NMR (75.5 MHz, $CDCl_3$):** δ 160.0, 154.6, 153.2, 134.8, 134.6, 130.1, 129.5, 129.2, 128.4, 120.8, 119.2, 117.1, 116.2 ppm.

Bibliography

[1] G. R. Fulmer, A. J. M. Miller, N. H. Sherden, H. E. Gottlieb, A. Nudelman, B. M. Stoltz, J. E. Bercaw, K. I. Goldberg, *Organometallics* **2010**, *29*, 2176–2179.

[2] N. R. Babij, E. O. McCusker, G. T. Whiteker, B. Canturk, N. Choy, L. C. Creemer, C. V. D. Amicis, N. M. Hewlett, P. L. Johnson, J. A. Knobelsdorf, F. Li, B. A. Lorsbach, B. M. Nugent, S. J. Ryan, M. R. Smith, Q. Yang, *Org. Process Res. Dev.* **2016**, *20*, 661–667.

[3] R. K. Harris, E. D. Becker, R. Goodfellow, P. Granger, *Pure Appl. Chem.* **2001**, *73*, 1795–1818.

[4] H. Li, S. Liu, Y. Huang, X.-H. Xu, F.-L. Qing, *Chem. Commun.* **2017**, *53*, 10136–10139.

[5] A. Mathuri, B. Pal, M. Pramanik, P. Mal, *J. Org. Chem.* **2023**, *88*, 10096–10110.

[6] A. Cervi, Y. Vo, C. L. L. Chai, M. G. Banwell, P. Lan, A. C. Willis, *J. Org. Chem.* **2021**, *86*, 178–198.

[7] C. Jia, D. Piao, T. Kitamura, Y. Fujiwara, *J. Org. Chem.* **2000**, *65*, 7516–7522.

[8] M. Roy, R. Jamatia, A. Samanta, K. Mohar, D. Srimani, *Org. Lett.* **2022**, *24*, 8180–8185.

[9] S. Sau, P. Mal, *Chem. Commun.* **2021**, *57*, 9228–9231.

[10] R. Pedrazzani, A. Pintus, R. De Ventura, M. Marchini, P. Ceroni, C. Silva López, M. Monari, M. Bandini, *ACS Org. Inorg. Au* **2022**, *2*, 229–235.

[11] Z. Wang, X. Li, L. Wang, P. Li, *Tetrahedron* **2019**, *75*, 1044–1051.

[12] M. D. P. Olaya, N. E. Vergel, J. L. López, D. Viña, M. F. Guerrero, *Braz. J. Pharm. Sci.* **2020**, *56*, e17609.

[13] C. Schultze, B. Schmidt, *J. Org. Chem.* **2018**, *83*, 5210–5224.

[14] Z. Shi, C. He, *J. Org. Chem.* **2004**, *69*, 3669–3671.

[15] D. Kim, M. Min, S. Hong, *Chem. Commun.* **2013**, *49*, 4021.
